# Precision diagnosis of burn injuries using imaging and predictive modeling for clinical applications

**DOI:** 10.1038/s41598-025-92096-4

**Published:** 2025-03-04

**Authors:** Pramod K. B. Rangaiah, B P Pradeep kumar, Fredrik Huss, Robin Augustine

**Affiliations:** 1 Microwaves in Medical Engineering Group, Division of Solid State Electronics, Department of Electrical Engineering, Uppsala University, Box 65, SE-751 03 Uppsala, Sweden; 2 Department of Computer Science and Design, Atria Institute of Technology, Bengaluru, 560024 India; 3 Department of Surgical Sciences, Plastic Surgery, Uppsala University, 751 05 Uppsala, Sweden; 4 Burn Center, Dept of Plastic and Maxillofacial Surgery, Uppsala University Hospital, Uppsala, Sweden

**Keywords:** Biomedical engineering, Image processing, Anatomy

## Abstract

Burns represents a serious clinical problem because the diagnosis and assessment are very complex. This paper proposes a methodology that combines the use of advanced medical imaging with predictive modeling for the improvement of burn injury assessment. The proposed framework makes use of the Adaptive Complex Independent Components Analysis (ACICA) and Reference Region (TBSA) methods in conjunction with deep learning techniques for the precise estimation of burn depth and Total Body Surface Area analysis. It also allows for the estimation of the depth of burns with high accuracy, calculation of TBSA, and non-invasive analysis with 96.7% accuracy using an RNN model. Extensive experimentation on DCE-LUV samples validates enhanced diagnostic precision and detailed texture analysis. These technologies provide nuanced insights into burn severity, improving diagnostic accuracy and treatment planning. Our results demonstrate the potential of these methods to revolutionize burn care and optimize patient outcomes.

## Introduction

Burn injuries pose a significant threat to life, resulting in severe morbidity and mortality^1^. These injuries present substantial challenges for the medical community, with complications such as organ failure, sepsis, and infection contributing to mortality rates ranging from 1.5% to 19%, and in severe cases, exceeding 35%^2^. This research introduces an advanced precision imaging technology, poised to significantly improve the accuracy of burn injury evaluation^3^. This innovative solution employs advanced deep neural networks to classify various burn levels within designated areas, surpassing conventional methods^4^. Such a sophisticated approach allows for a more comprehensive assessment of skin status and burn severity^5^.

The World Health Organization recommends at least one burn unit bed per 6,000,000 people, emphasizing the seriousness of burn injuries^5,6^. However, the geographical separation of burn centers and reliance on non-specialized professionals for diagnosis pose significant challenges^7^. Our proposed deep neural network classification method covers categories including healthy skin, superficial burn (epidermal, first degree), superficial dermal burn (second degree (a)), deep dermal burn (second degree (b)), and full-thickness burn (third degree). Each category represents a distinct level of tissue damage, enabling a detailed evaluation of burn severity^8^. In cases where a specific region exhibits features inconsistent with a single burn type, the deep neural network employs advanced techniques to determine the most pertinent degree of burn dynamically^9^. These algorithms enhance the precision and consistency of burn injury classification, providing clinicians with accurate and comprehensive information necessary for well-informed decisions^10^.

Accurate assessment of burn injuries, including their depth and affected surface area, is critical for determining appropriate treatment strategies. Traditional methods often rely on subjective evaluations by clinicians, leading to variability and inconsistencies in diagnosis. This variability underscores the need for objective, standardized tools^11^ capable of delivering reliable and reproducible results. In this context, we introduce “SenseBurn”, a novel diagnostic tool developed as part of our collaborative research efforts to address these clinical challenges. SenseBurn is a non-invasive diagnostic system designed to analyze burn injuries with high precision by integrating advanced imaging techniques and computational models. The tool quantifies burn depth and Total Body Surface Area (TBSA) through an automated framework, combining real-time imaging with machine learning-based classification. By leveraging these advanced methodologies, SenseBurn provides objective assessments of burn severity, mitigating the inconsistencies inherent in traditional methods.

The development of SenseBurn was supported by research initiatives such as Eurostars and Vinnova, which facilitated the integration of cutting-edge technology into clinical burn diagnostics. The project’s aims include enhancing diagnostic accuracy, improving clinical decision-making, and optimizing patient outcomes. Extensive validation in both research and clinical settings has demonstrated the potential of SenseBurn to transform burn evaluation and treatment planning. Additional information about the project is available through the SenseBurn website^12^ https://www.senseburn.com/, Vinnova’s project description^13^ https://www.vinnova.se/en/p/e12052---senseburn/, and the Eurostars overview^14^.

This work investigates the practical implications of incorporating Adaptive Complex Independent Components Analysis (ACICA) and reference region (RR) technologies into clinical practice, emphasizing their potential to transform burn injury assessment and management. This system aims to enhance treatment planning, improve patient outcomes, and contribute substantially to advancing burn care practices by providing a more detailed understanding of burn depths.

**Fig. 1:**
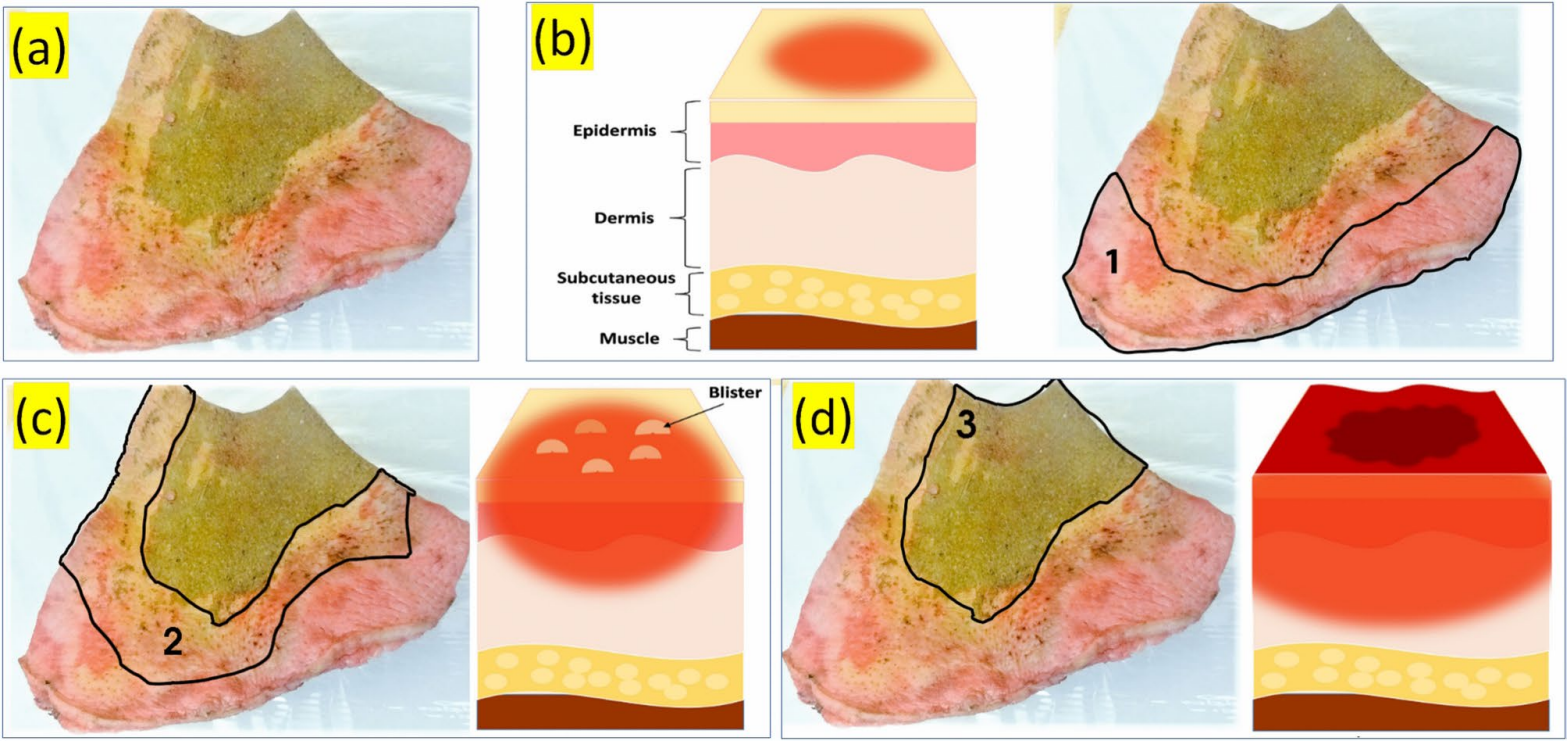
Graphic illustrating the varying degrees of burns: (**a**) Burn sample; (**b**) Superficial burn; (**c**) Superficial and deep dermal burn, with sketch C representing primarily a superficial dermal burn; and (**d**) Full-thickness burn, with sketch D depicting a deep dermal burn. For a true full-thickness burn, the red area should extend beneath the dermis into the subcutaneous fat.

Figure 1(a) depicts a specimen sample taken from a human burn injury, providing a valuable tool for exploring cellular and molecular changes during the healing process. This examination facilitates understanding the extent of the burn and investigating potential treatment options. Figure 1(b) highlights a superficial burn, where damage is confined to the epidermis, the outermost skin layer. Superficial burns generally heal within 2–3 weeks without scarring, although they may lead to hypo- or hyperpigmentation. Including this burn type in the study aids in investigating the early stages of burn injuries and identifying factors that influence full recovery. These various burn examples collectively contribute to a thorough understanding of burn injuries and their treatment, enhancing the precision of burn assessment and improving clinical outcomes

Figure 1(c) shows a superficial and deep dermal burn sample, allowing for the examination of the burn’s superficial and deep dermal layers. Understanding the degree of damage in these layers is crucial for developing appropriate treatment plans. The research focuses on comparing the healing processes and responses associated with these different burn injury layers. Figure 1(d) illustrates a full-thickness burn, showing the damage that penetrates the entire thickness of the skin, encompassing the epidermis, dermis, and potentially even deeper underlying tissues. Such burns typically require specialized medical care due to their severity. This work includes this burn type to explore the challenges and mechanisms associated with deep-tissue burn healing.

### Objective of the study

This work addresses a significant gap in medical imaging processing and diagnostics by focusing on the precise estimation and identification of burn characteristics. One of the major challenges is developing a reliable burn area identification technique without relying on dynamic contrast enhancement (DCE). The absence of DCE can reduce the sensitivity and specificity of burn assessments, affecting the reliability of distinguishing burn profiles.

To overcome this, our system incorporates dynamically enhanced burn samples. These dynamic images capture contrast agent distribution and temporal variations in tissue perfusion, providing crucial insights into tissue characteristics. We utilize the ACICA approach to analyze these enhanced images effectively. ACICA is known for its ability to interpret complex data relationships, enhancing our capacity to distinguish subtle differences in burn profiles.

By integrating dynamically enhanced burn samples with ACICA, our method improves sensitivity and specificity in burn depth identification. These metrics highlight the framework’s potential for early diagnosis and prompt intervention. We explore the distributional properties and features of burn-enhancing agents using parameters such as entropy, skewness, and kurtosis. These metrics provide a comprehensive understanding of tissue complexity, further enhancing the accuracy of burn assessment and management.

The paper makes significant contributions to the field of burn analysis through several innovative approaches. The LUV color space, where ’L’ represents luminance, and ’U’ and ’V’ represent chrominance components, is used for separating brightness from color information, thereby enabling more precise analysis of tissue variations in burn injuries. Firstly, it introduces a groundbreaking method by utilizing dynamic contrast enhancement in transformed RGB to LUV images. This novel approach combines in-depth pixel-level classification, accurate region identification via RR, and automated color correction using ACICA. Together, these techniques form a comprehensive and efficient method for evaluating burn injuries. Furthermore, the paper emphasizes the importance of personalized care through accurate diagnosis. By offering precise assessments of burn area and depth, the proposed approach enables tailored treatment plans in the early stages of burn injuries. This is achieved through the integration of non-invasive burn-depth analysis, burn area identification, and a deep learning network trained on clinical data, advancing towards personalized and patient-specific treatment techniques^15^.

The paper facilitates a comprehensive assessment of burn severity. Through the deep neural network’s categorization system, which spans from healthy skin to full-thickness burns, physicians gain valuable insights into the degree of tissue damage. This sophisticated categorization system aids in making informed decisions regarding optimal treatment strategies based on the severity of the burn injury. Moreover, the paper demonstrates adaptive categorization for diverse burn characteristics. The deep neural network’s adaptive method enhances the accuracy and reliability of burn injury categorization by intelligently selecting the most appropriate degree of burn. This flexibility allows the system to manage diverse burn characteristics effectively, even in situations where certain regions exhibit features inconsistent with a single type of burn.

By integrating RR and ACICA for accurate region recognition and automated color correction, this work advances burn analysis methodologies significantly. This comprehensive integration underscores a multifaceted approach to burn assessment, providing professionals with an effective tool for rapidly and reliably diagnosing and treating burn injuries. This work makes the following key contributions:

- Precision Burn Depth Estimation: The integration of ACICA and RR methods provides accurate burn depth quantification, surpassing the limitations of traditional visual assessments, thus improving diagnostic reliability.
- Innovative Imaging Techniques: Dynamic Contrast Enhancement (DCE) combined with GLCM-based texture analysis delivers detailed tissue characterization, facilitating differentiation between various burn types.
- Predictive Modeling Integration: Advanced machine learning models (CNN, FNN, RNN) significantly enhance the prediction of burn severity, enabling informed and timely clinical decisions.
- Enhanced TBSA Analysis: A novel approach to Total Body Surface Area (TBSA) estimation ensures a detailed and precise understanding of burn severity, aiding in effective treatment planning.
- Clinical Utility: By linking computational advancements with clinical needs, the proposed system enhances patient outcomes through accurate diagnosis and tailored, effective treatment strategies.

The organizational structure of the research article reflects a systematic approach to presenting these contributions. Section II, “Related Works,” offers a detailed survey of previous research, focusing on image enhancement methods and their applications. In Section III, “Methods,” a unique image enhancement framework tailored specifically for DCE-burn samples is introduced. Experimental results, employing quantitative evaluations and visual comparisons to validate the framework, are discussed in Section IV. Finally, Section V: Conclusion, summarizes key findings and offers recommendations for further study.

## Related works

Burn injury management relies heavily on accurate assessment of the total body surface area (TBSA) affected and the depth of the burn wounds to determine appropriate treatment strategies. Jeschke et al. (2020) highlighted the challenges in visually determining burn depth, especially in distinguishing between superficial, dermal burns that may not require surgical intervention and deeper burns necessitating grafting. This underscores the importance of measurement tools for precise assessment^16^.

Various non-invasive techniques have been explored to extract precise information about burn depth and severity. These include Laser Doppler Imaging (LDI), thermography, video microscopy, orthogonal polarization spectral imaging (OPSI), reflectance confocal microscopy (RCM), multispectral imaging (MSI), optical coherence tomography (OCT), near-infrared spectroscopy (NIRS), terahertz imaging, laser speckle imaging (LSI), spatial frequency domain imaging (SFDI), photoacoustic imaging, and ultrasound.

Martins et al.^17^(2022) compared various imaging systems and found Laser Doppler Imaging (LDI) to be reliable, while others such as infrared thermography or spectrophotometric intracutaneous analysis were less accurate^18^. However, many of these systems face limitations such as high cost, intolerance to patient movement, and long scanning times, except for ultrasound and photoacoustic imaging^17^. There is a need for standardized protocols for effective quantification of burn depth, as highlighted by the work on conformal sensors. Modern classification techniques categorize burn degrees into superficial, deep partial, deep dermal, and full-thickness burns. Żwierełło et al. (2023) emphasized the significance of accurately distinguishing burn severity and depth to determine appropriate treatment, with deep dermal and full-thickness burns often requiring surgical debridement and skin grafting^19^. Clinical evaluation remains the primary method for assessing burn depth, but its accuracy is estimated to be up to 70%, with significant variability between assessors^20^. Consistent diagnostic data is crucial for effective medical interventions, as the distribution and depth of the burn determine the severity of the injury and the level of care required^21^.

Recent studies have demonstrated the efficacy of color space transformations in medical imaging. The transformation from RGB to LUV color space, for instance, provides a more perceptually meaningful representation of colors, which is crucial for accurate burn analysis. The LUV color space separates luminance from chrominance, allowing for precise detection of tissue color variations indicative of different burn severities. Li et al. (2023) demonstrated that LUV transformation^22^enhances the contrast between burn and healthy tissue, facilitating more accurate segmentation and analysis^23^.

Dynamic Contrast Enhancement (DCE) techniques have been pivotal in burn imaging research. DCE enhances the visualization of blood flow and vascularity in burn tissues, providing critical information about burn depth and severity. By applying contrast agents, DCE highlights areas with increased perfusion, often correlated with more severe burns. Studies by Dong et al. (2021) confirmed that DCE improves diagnostic accuracy by providing detailed vascular maps of burn injuries^24^.

ACICA has emerged as a method for accurately quantifying contrast agents in medical imaging. ACICA addresses challenges in early-stage contrast agent uptake, offering a reliable measure of tissue perfusion and burn severity. Recent advancements by Setia et al. (2024) demonstrated that ACICA significantly enhances the quality of medical image analysis and diagnosis by effectively isolating and quantifying contrast agents^25^. The RR approach has been explored for its ability to correlate imaging characteristics^26^with burn severity. The RR method allows for statistical analysis of imaging data, providing a framework for segmenting grayscale images by intensity clusters. Research by Kotaridis et al. (2023) showed that RR-based segmentation improves the precision of burn severity assessments by clearly delineating burn regions based on contrast uptake^27^.

Texture analysis using the Grey Level Co-occurrence Matrix (GLCM) is an effective technique for differentiating tissue types and structural changes in burn injuries. GLCM-based methods extract important texture features such as contrast and correlation, essential for accurate segmentation and classification of burn regions. Research by Pabitha et al. (2024) highlighted the use of GLCM in enhancing the differentiation between superficial, dermal, and full-thickness burns^28^.

Machine learning models, including Recurrent Neural Networks (RNN), Feedforward Neural Networks (FNN), and Convolutional Neural Networks (CNN), have been extensively applied in medical image analysis. These models classify and predict burn severity based on extracted features from large datasets. Hu et al. (2023) demonstrated that CNNs provide high accuracy in burn classification and TBSA estimation due to their ability to learn complex patterns from image data^29^.

The integration of advanced imaging and computational techniques represents a significant advancement in burn injury assessment. Combining color space transformations, DCE, ACICA, RR methods, GLCM-based texture analysis, and machine learning models have resulted in comprehensive frameworks that provide detailed and accurate evaluations of burn severity and distribution. The work by Zhong et al. (2023) exemplifies this integration, resulting in a robust system that enhances diagnostic precision and treatment planning^30^.

**Table 1:** Comparison of Advanced Imaging Modalities for Burn-Depth Analysis Detection and Assessment.

**Imaging Modality**	**Advantages**	**Clinical Applications**	**Limitations**
Infrared Thermography	Non-invasive and real-time monitoring, Rapid, non-contact imaging^31^	Early diagnosis of burn depth and progression, Monitoring treatment response and wound healing	Limited penetration depth, Sensitivity to ambient temperature changes introduces variability
Multispectral Imaging	Enhanced tissue contrast with multi-wavelength data, Detailed imaging for vascular changes	Identification of burn severity and tissue differentiation^32^, Assessment of vascular changes in burn wounds	Limited availability, Potential depth limitations
Magnetic Resonance Imaging (MRI)	Superior soft tissue contrast and multiplanar imaging^33^, Differentiation of burn depth from adjacent structures	Detailed characterization of burn injuries, Differentiation of burn depth from adjacent structures	Limited availability, especially in acute or critical settings^34^, Longer acquisition times may pose challenges
Ultrasound^35^	Real-time imaging and cost-effectiveness, Widely accessible	Superficial and deep burn depth estimation, Monitoring changes in burn injuries over time	Operator dependence, Suboptimal visualization of deeper structures
Optical Coherence Tomography (OCT)^36^	High-resolution imaging and non-invasiveness, Early detection of changes in burn depth	Microstructural assessment of burn injuries, Early detection of changes in burn depth	Limited penetration depth, Limited field of view
Thermal Imaging^37^	Non-contact and rapid data acquisition, Identification of variations in burn depth	Monitoring temperature distribution in burn areas, Identification of variations in burn depth	Lower spatial resolution, Sensitivity to environmental factors
Computed Tomography^38^	Detailed vascular assessment and 3D imaging for surgical planning	Identification of perfusion abnormalities in severe burns	Limited soft tissue contrast
Angiography (CTA)^39^	Guidance for surgical interventions in severe burn cases	Guidance for surgical interventions in severe burn cases	High cost
Photoacoustic Imaging	Functional and molecular information with non-ionizing radiation^40^, Research into early detection of burn-related complications	Improved diagnostic accuracy in burn depth assessment, Research into early detection of burn-related complications	Limited depth penetration in highly pigmented tissues, Equipment complexity and cost may impact accessibility

Table 1shows a comprehensive comparison of advanced imaging modalities used for burn-depth analysis, detailing their advantages, clinical applications, and limitations. Burn injuries pose a serious threat to life and present considerable challenges for the medical community. These injuries can lead to severe complications, including organ failure, sepsis, and infection, which can significantly impact mortality rates. The severity of burns can vary widely, with mortality ranging from minimal to critical levels, depending on the extent and depth of the injury. To address these critical issues, our study introduces a novel precision imaging technology that aims to revolutionize the assessment of burn injuries^41^. This innovative approach employs advanced deep neural networks to classify various burn levels within designated areas, surpassing conventional methods. Such a sophisticated understanding allows for a comprehensive assessment of skin health and burn severity.

Infrared Thermography is a non-invasive and real-time imaging technique useful for burn-depth analysis. It is particularly effective for early identification of burn depth and progression, and monitoring treatment response and wound healing^42^. However, its limited penetration depth may hinder detailed insights into deeper tissue layers, and its sensitivity to ambient temperature changes can introduce variability. Despite these limitations, thermography is frequently used in initial burn assessments due to its ability to quickly and non-invasively capture surface temperature variations^43^.

Multispectral Imaging provides enhanced tissue contrast and multi-wavelength data, allowing for detailed imaging necessary to determine burn severity and tissue differentiation^44^. Clinically, it is used to assess vascular changes in burn wounds^45^. However, limited availability and potential depth limitations can impact its accessibility. Its ability to offer fine-grained tissue contrast makes it valuable for detecting different burn levels and tissue types^46^.

Ultrasound is well-known for its affordability and real-time capabilities, making it widely accessible^47^. It is used to estimate the depth of both superficial and deep burns and to monitor changes in burn injuries over time^48^. However, its effectiveness can be limited by operator dependence and suboptimal visualization of structures. Ultrasound remains a preferred method for quick assessment of burn injuries due to its cost-effectiveness and real-time imaging^49^.

Optical Coherence Tomography (OCT) offers high-resolution, non-invasive imaging for early detection of changes in burn depth and microstructural assessment of burn injuries^50^. However, its shallow penetration depth and limited field of view restrict its applicability to fine-grained, surface-level structural visualization^51^.

Thermal Imaging is a valuable tool for tracking changes in burn depth and temperature distribution in affected regions due to its quick data capture and non-contact nature^52^. However, its lower spatial resolution and susceptibility to environmental factors are notable limitations. It is often used in real-time analysis of temperature distribution in burn areas^53^.

Computed Tomography Angiography (CTA) provides detailed vascular assessment and 3D imaging, making it essential for diagnosing perfusion anomalies in severe burn patients and guiding surgical procedures^54^. However, its limited soft tissue contrast and high cost must be considered. CTA is frequently selected for cases requiring thorough vascular evaluation and surgical planning in severe burns^55^.

Photoacoustic Imaging combines functional and molecular information with non-ionizing radiation, improving diagnostic accuracy in burn depth assessment and advancing research into early detection of burn-related complications^56^. However, its limited depth penetration in highly pigmented tissues and equipment complexity can impact accessibility^57^. Photoacoustic imaging is preferred for its potential to enhance diagnostic accuracy through functional and molecular insights^58^.

The related work highlights the continuous evolution and innovation in burn injury assessment methodologies. Advancements in imaging techniques, computational models, and machine learning collectively contribute to more accurate and reliable diagnosis and treatment planning for burn patients. Our study builds on these foundational works, aiming to further enhance the precision and comprehensiveness of burn injury evaluations through the integration of state-of-the-art methods and cutting-edge technologies.

## Methods

This section describes the methodology that was used to develop and validate the proposed framework in diagnostics related to burn injuries. The proposed framework combines state-of-the-art computational imaging techniques with predictive modeling for precise assessment of burn profiles. It includes key processes such as RGB-to-LUV image transformation for better color-space representation, DCE to enhance the visibility of critical features, and advanced texture and statistical analysis methods like GLCM. These steps, together with deep learning models, provide a robust system for accurate classification of burn severity and estimation of the total body surface area.

### Ethical approval and consent

Approval for this study was granted by the Regional Ethics Committee in Uppsala, Sweden, and the research was conducted by the “Ethical Principles for Medical Research.” Informed consent discussions were held with all participants before any study procedures. Ethical approval was sought from the Swedish National Ethical Committee under the framework of the EUREKA Eurostars SenseBurn project on non-invasive burn depth assessment. Permission for the study was granted by the Regional Ethical Review Board in Uppsala (Dnr 2018/410), tracked by biobank and hospital approvals on February 1st, 2019.

Participating patients were provided with comprehensive information about the study and gave written informed consent for participation. In cases where participants were initially incapable of providing consent, written consent was obtained from the patient’s next of kin and subsequently from the participants themselves when feasible. The study adhered to the International Conference on Harmonisation - Good Clinical Practice (ICH-GCP) guidelines, the Helsinki Declaration, and its subsequent revisions. Additionally, the time between excision and ex-vivo assessments was minimized to prevent tissue drying and ensure accurate measurements.

### Data acquisition

All RGB images of burn patients were acquired at Uppsala University, Sweden. Images were captured in JPEG format using a camera positioned approximately 30–50 cm from the burn wound without using a flash. This setup ensures consistent image quality and minimizes variations caused by external lighting.

To ensure the integrity of the tissue samples, efforts were made to prevent fluctuations in water content and temperature. Tissues were transported in hermetically sealed containers to maintain consistent temperature conditions. Samples of burned human skin, ranging from full-thickness to superficial burns, were collected from surgical waste at the University Hospital’s Burn Centre in Uppsala, Sweden. The research adhered to relevant national ethical guidelines and regulations. The samples were sourced from various areas of the human body, and the University Hospital’s Burn Centre meticulously maintained metadata. Subsequently, the samples were transported to the microwave lab at the Ångström Laboratory, Uppsala University, Sweden. Throughout this process, a measurement form was maintained, documenting the clinical assessment^59^ of all samples to ensure accurate and reliable data collection.

**Fig. 2:**
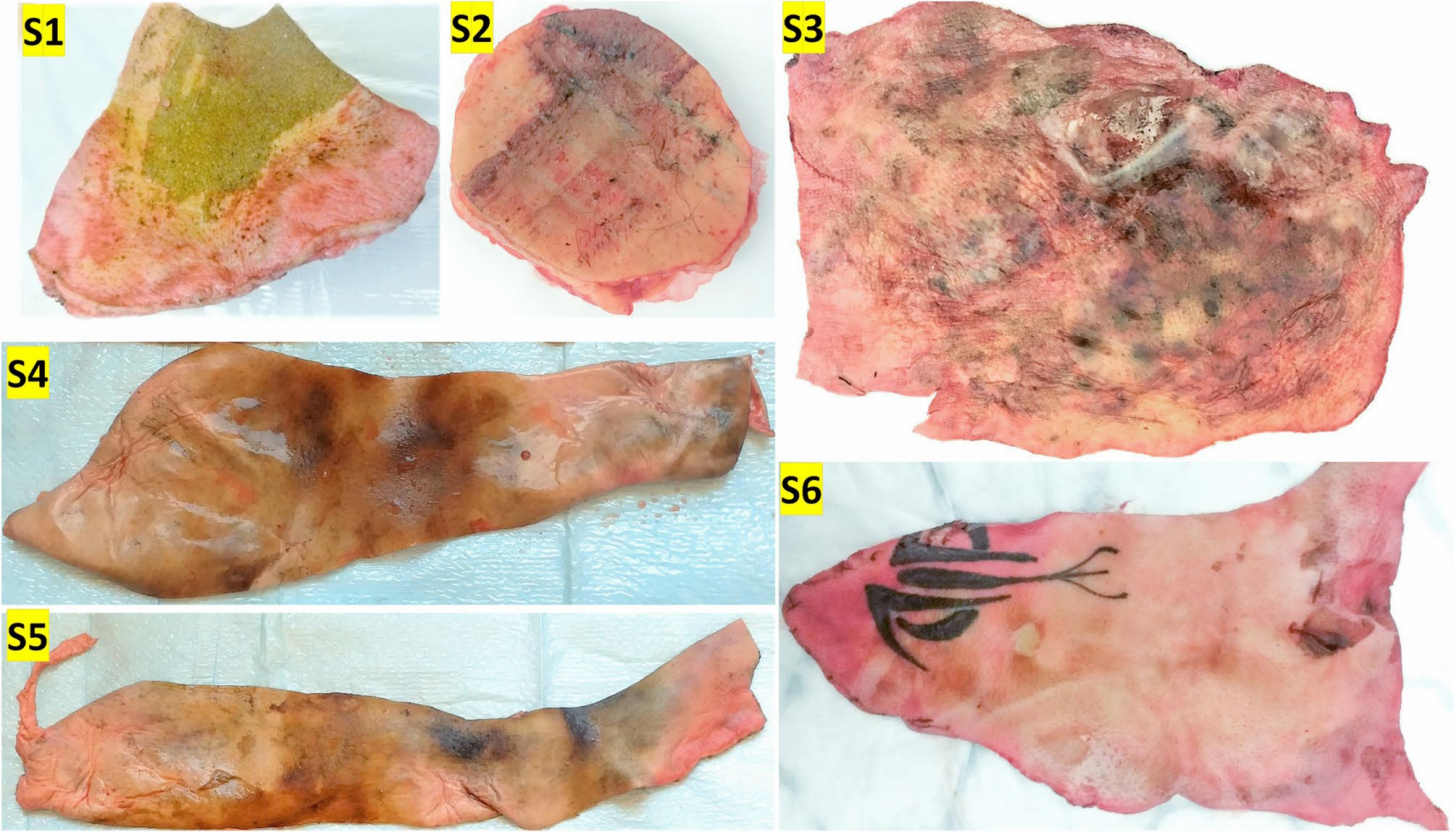
Original Images of Burn Samples (S1-S6).

Figure 2 displays the original images of six burn samples (S1-S6) used in this study. Each image serves as the foundational data for subsequent image processing and analysis techniques. The samples exhibit a range of burn severities, with varying degrees of superficial, superficial dermal, and deep dermal tissue damage. These images were captured under standardized conditions to ensure consistency and reliability in the analysis. The diversity in the burn patterns across the samples provides a robust dataset for testing and validating the advanced diagnostic methodologies employed in this research.

In the data acquisition process, high-resolution images of the burn samples were obtained using a standardized imaging setup to ensure uniform lighting and color consistency. These original images are critical for the initial assessment and serve as the input for further processing steps, including color space transformation, texture analysis, and segmentation techniques. The captured images encompass various burn depths and patterns, providing a comprehensive dataset that reflects real-life clinical scenarios. This diversity is essential for developing and testing robust diagnostic frameworks capable of accurately assessing different types of burns. The quality and detail in these original images are pivotal for the success of subsequent analytical methods, ensuring that the extracted features are both accurate and representative of the actual burn injuries.

### Experimental setup

The experimental setup was meticulously designed to evaluate the proposed framework for burn injury assessment, ensuring robust and reproducible results. All experiments were conducted on a high-performance workstation equipped with an Intel Core i7 (12th Gen) processor, NVIDIA RTX 3080 GPU with 10 GB VRAM, 32 GB DDR4 RAM, and a 1 TB SSD for efficient data processing and model training. The implementation was carried out using MATLAB R2022b, integrated with Python-based TensorFlow libraries for machine learning tasks.

A dataset comprising 90 clinically annotated burn sample images was used for the experiments. The dataset was stratified into training (70%), validation (15%), and testing (15%) subsets to maintain a balanced representation across burn depth categories. Each image was preprocessed by resizing to $224 \times 224$ pixels, normalizing intensity values to a range of [0, 1], and applying data augmentation techniques, including random rotation ($\pm 15^\circ$), flipping, and brightness adjustments ($\pm 20\%$), to enhance generalization and prevent overfitting. This systematic setup ensured the reliability and scalability of the proposed methodology, addressing challenges in burn injury assessment and enabling precise classification and analysis of burn depths.

### Burn depth assessment survey

SenseBurn is developing a non-invasive microwave sensor-based diagnostic tool aimed at enhancing the accuracy and efficiency of burn analysis. This tool is designed to precisely diagnose burn depth and area, facilitating immediate and appropriate treatment. SenseBurn comprises three innovative components: a non-invasive microwave burn-depth sensor, 3D burn area imaging, and intelligent software that estimates burn profiles based on clinical trials. This tool promises to be fast, cost-effective, portable, and versatile.

Currently, burn depth and extent are primarily assessed through clinical evaluation, which is scientifically proven to be only about 70% accurate, with significant variability between assessors. To address these inconsistencies, we conducted a survey to highlight the challenges and discrepancies in visual clinical assessments of burn wounds. Clinicians were presented with high-resolution images of excised allogenic burned skin samples and asked to evaluate burn depth in various areas.

The skin samples used in this survey were obtained from surgical waste, specifically tissue removed during burn treatments at the Burn Center, Uppsala University Hospital, and normal skin excised during plastic surgery procedures. Instead of being discarded, the excised tissue was transported to a laboratory for measurement and histological processing.

Patients admitted to the Burn Center and the Department of Plastic and Maxillofacial Surgery met the inclusion criteria and were approached for informed consent. Patients undergoing burn or plastic surgery involving tissue removal were asked for permission to transport samples to the laboratory for measurement. Tissue samples were also sent for histological processing, staining, and assessment before being discarded. Clinicians were asked to review the images and mark areas corresponding to the following burn depths: A: Healthy skin; B: Superficial burn; C: Superficial dermal burn; D: Deep dermal burn, and E: Full-thickness burn

#### EBA 2019 survey

Initially, a hard copy survey for visual burn wound assessment was prepared as a handout at the European Burns Association Congress (EBA) 2019 in Helsinki. However, this approach was unsuccessful due to insufficient feedback.

#### Survey among German burn surgeons

In response to the lack of completed questionnaires, we visited burn centers in Germany to collect feedback directly from clinicians using a tablet computer (iPad). This method proved effective, as clinicians could draw and mark images using an electronic pen (Apple Pencil) and correct their inputs as needed. However, it also posed a challenge, as groups of burn surgeons often discussed samples together and completed only one questionnaire. The COVID-19 pandemic further restricted access to hospitals, necessitating an alternative approach via an online survey using Google Forms.

#### Online survey using google forms

Google Forms, a tool for creating online surveys and questionnaires, was used to collect and organize information. Responses were automatically stored and analyzed within Google Sheets. The survey focused on understanding the challenges of visually inspecting burned skin using high-resolution images of excised allogenic burned skin samples. These images were divided into areas with different burn depths using Adobe Photoshop$\circledR$. Compliant with the General Data Protection Regulation (GDPR), the questionnaire was anonymous, and no personal data was collected. Participants were instructed: “Please choose the corresponding skin condition or degree of the burn. If a specific area does not match a single type of burn, choose the degree that mainly corresponds.”

Given the limitations of clinically inspecting excised cadaveric skin samples in an online survey, factors like temperature, capillary reaction, edema, and damage to skin appendages were not considered. The survey was rolled out on 15.07.2021 among burn surgeons and members of the European Burns Association, with 27 participants completing it between 15.07 and 28.07.2021. The variance in evaluations by different participants highlighted the individual differences in estimating burn depth. For example, sample 04-2 had 100% agreement for healthy skin, while sample 02-1 showed significant discrepancies in assessment.

**Fig. 3:**
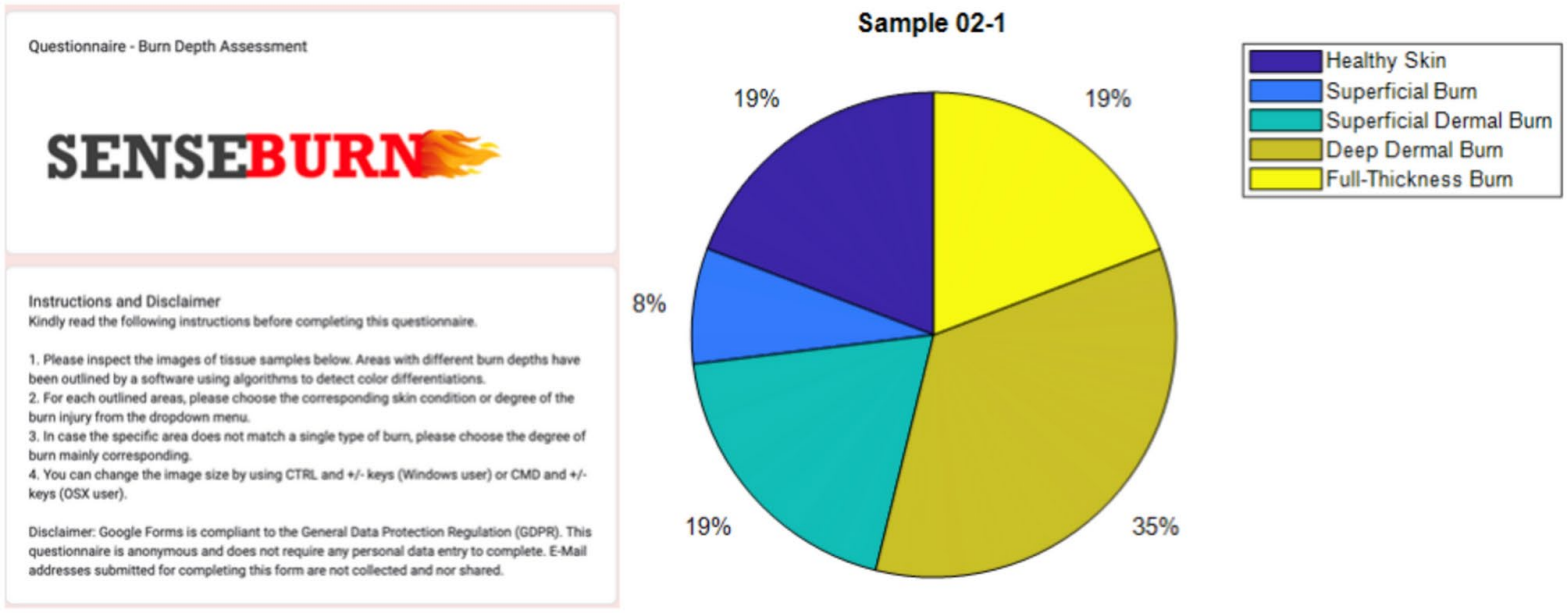
Distribution of skin conditions and burn depths in Example: Sample 02-1: Healthy Skin (19.2%), Superficial Burn (7.7%), Superficial Dermal Burn (19.2%), Deep Dermal Burn (34.6%), Full-Thickness Burn (19.2%).

Figure 3 illustrates the proportion of different skin conditions and burn severities in Sample 02-1. Each segment of the pie chart represents a specific condition: Healthy Skin (19.2%), Superficial Burn (7.7%), Superficial Dermal Burn (19.2%), Deep Dermal Burn (34.6%), and Full-Thickness Burn (19.2%). This visualization helps in understanding the distribution of burn injuries within the sample. Our survey demonstrated the challenges in the visual inspection of burned skin and provided insights into the variability of clinical assessments. This underscores the need for novel diagnostic tools for burn wound assessment.

Accurate clinical assessment of burn injury often depends on subjective observations by clinicians, which introduces great variability into diagnoses and treatments. In order to identify limitations in current practices, we conducted a survey among clinicians and researchers. The survey was designed to capture their perceptions about the reliability, consistency, and practicality of existing methods for assessing burn depth and extent.

The most salient findings of the survey process were inconsistent feedback among respondents because of variable levels of clinical expertise and subjective assessments, which had to be matched to objective metrics. These findings relate directly to the clinical evaluation of burns because they reflect limitations inherent in subjective methods of assessment alone, which can result in diagnostic variability and suboptimal treatment. These findings underscore the need for robust, objective tools such as SenseBurn that seek the standardization of burn injury assessment, integrating advanced imaging and computational techniques. By tackling the inconsistencies in the survey, the proposed framework tries to reduce subjectivity and thus enhance the accuracy, reproducibility, and decision-making for diagnosis and clinical treatments. This thereby links the outcomes of the survey with the development of SenseBurn by reinforcing that addressing survey difficulties does have real relevance to broader clinical burn evaluation.

### Proposed framework

**Fig. 4:**
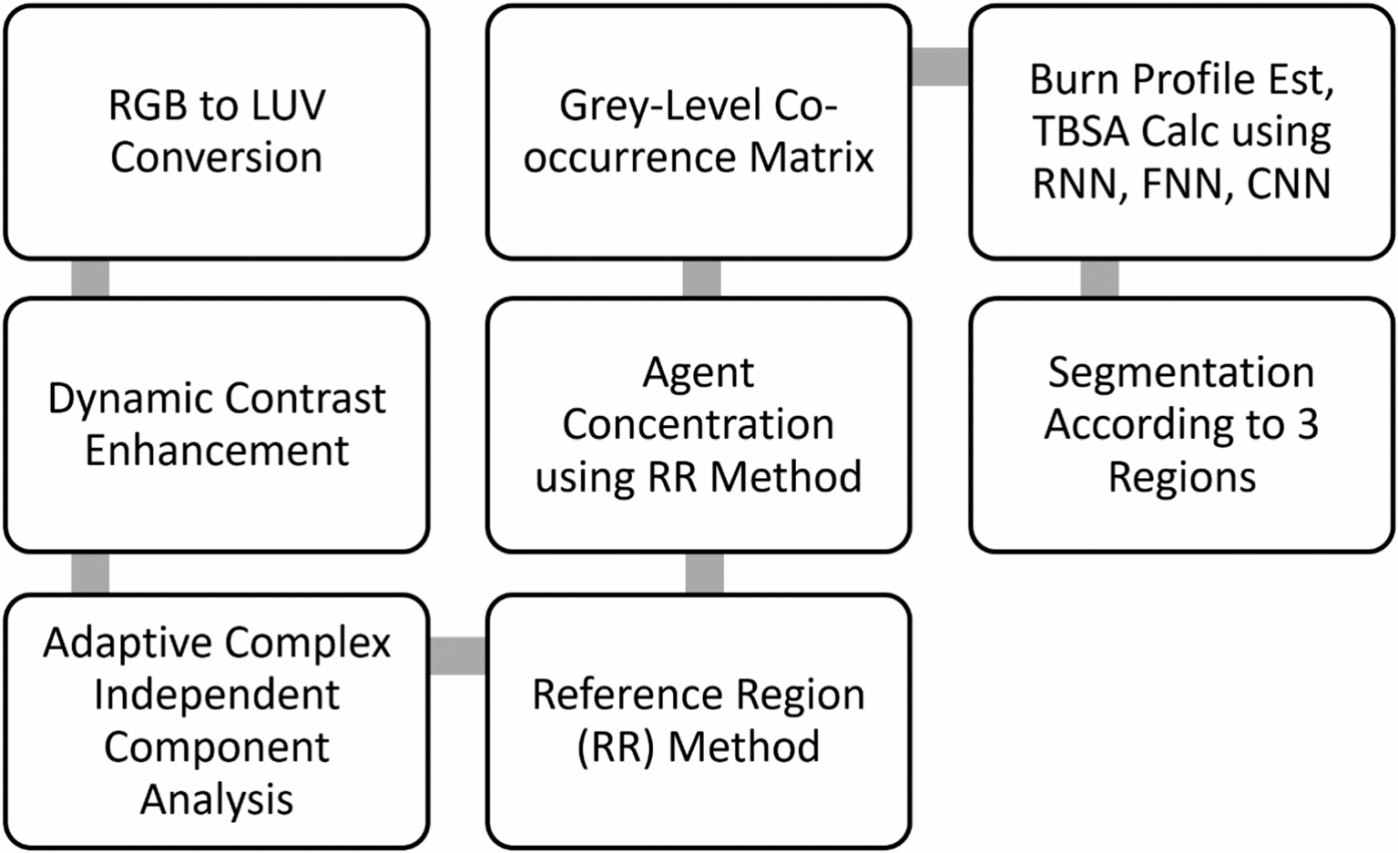
Architectural Blueprint for Enhanced Burn Profile Diagnosis and Estimation.

The proposed methodology integrates advanced computational techniques and neural network models to create a robust framework for burn injury diagnosis and classification. The process begins with the conversion of RGB images to the LUV color space, which enhances image analysis by separating luminance (brightness) from chrominance (color information). This transformation improves the precision of subsequent image processing steps. Dynamic Contrast Enhancement (DCE) is applied next to improve the visibility of burn regions, making it easier to extract critical features and assess burn severity. Figure 4 illustrates the architectural blueprint for a sophisticated burn injury diagnostic system, integrating advanced computational imaging and predictive modeling techniques to accurately assess burn profiles. The process begins with the transformation of RGB color space images to LUV color space, which enhances color analysis by separating luminance from chrominance. This transformation is crucial for improving the precision of subsequent image analyses. Following this, Dynamic Contrast Enhancement (DCE) is applied to the burn sample. DCE leverages contrast agents to highlight critical information on burn severity. This step enhances the visibility of burn regions, facilitating the extraction and analysis of important features.

The next step involves the ACICA method, which is employed to accurately quantify contrast compounds. ACICA is particularly effective in deciphering complex data relationships, making it a powerful tool in medical imaging and diagnostics. This method significantly contributes to the precise quantification of tissue characteristics. The reference Region (RR) method is utilized to establish the relationship between imaging characteristics and burn severity, enabling comprehensive statistical analysis of the data. The RR-Method segments images into intensity clusters, resulting in an Agent Concentration Image. This segmentation is essential for a focused and precise analysis of burn severity and extent. the percentage of the contrast agent is calculated using the RR method, aiding in the quantification of tissue perfusion, a critical indicator of burn depth.

Features such as texture information are then derived by employing the Grey-Level Co-occurrence Matrix. The Grey Level Co-occurrence Matrix (GLCM) is a statistical approach for extracting texture features by evaluating the spatial dependencies of pixel intensities. In this paper, the GLCM features include contrast: the result of high-intensity variations between contiguous pixels, helping in the detection of irregular patterns associated with the burn lesion. Correlation: Indicates texture homogeneity, reflecting structural consistency within tissue regions. These features are highly useful in the differentiation between superficial, dermal, and deep burns because they quantify texture differences that correspond to different degrees of tissue damage. By closing the loop between raw image data and clinically actionable information, GLCM-based texture analysis significantly improves the accuracy of burn segmentation and classification to support diagnosis and treatment planning with precision.

The extracted features are then fed into advanced neural network models, including Recurrent Neural Networks (RNN), Feedforward Neural Networks (FNN), and Convolutional Neural Networks (CNN). These models estimate the burn profile and calculate the Total Body Surface Area (TBSA) affected by the burn. The use of these sophisticated models ensures accurate and reliable diagnostic outcomes, classifying and predicting burn severity based on the extracted features.

For a more detailed investigation, the burn area is segmented into three regions: superficial, dermal, and deep dermal burns. This segmentation is based on the thorough analysis and feature extraction steps, allowing for precise classification and diagnosis of burn severity across different tissue layers. This comprehensive approach ensures an accurate and precise diagnosis, aiding clinical decision-making and optimizing treatment outcomes for burn patients.

The framework introduces an innovative technique that employs efficient article abstraction methods based on descriptors, aimed at enhancing the outcomes of burn image profile estimation. Implemented within the MATLAB environment, the framework is structured around burn profile estimation. This ongoing refinement and innovation underscore the commitment to advancing the accuracy and efficacy of burn profile diagnosis and estimation methodologies.

#### Computational techniques

The computational techniques employed in this study include ACICA, the GLCM, and a CNN. Each of these methods plays a vital role in analyzing burn images to determine region, depth, and total body surface area (TBSA). ACICA is a statistical method designed to separate mixed signals into their independent components. In this study, ACICA was utilized to enhance the analysis of contrast agent distribution in burn images, particularly in distinguishing complex tissue structures. The method is based on the following relationship:

1 $$\begin{aligned} \textbf{X} = \textbf{A} \cdot \textbf{S}, \end{aligned}$$

where $\textbf{X}$ represents the observed data matrix, $\textbf{A}$ is the mixing matrix, and $\textbf{S}$ denotes the independent source signals. ACICA optimizes the unmixing matrix $\textbf{W}$, where $\textbf{W} = \textbf{A}^{-1}$, to reconstruct independent components from observed data. By minimizing mutual information between components, ACICA isolates regions of interest and addresses noise and complex tissue structures, ensuring precise delineation of burn regions and depths. The GLCM is employed to extract texture features from grayscale images, quantifying the spatial relationships between pixel intensities. The CNN employed in this study is designed for the classification of burn depths. The network processes input images resized to $224 \times 224$ pixels, extracting spatial features such as edges and textures through convolutional layers. The CNN architecture includes three convolutional layers with $3 \times 3$ kernels, followed by Rectified Linear Unit (ReLU) activation. The number of filters increases from 32 in the first layer to 64 and 128 in subsequent layers, enhancing the model’s capacity to capture complex features. Max-pooling layers with $2 \times 2$ kernels are used to downsample feature maps, while dropout layers with a rate of 0.25 prevent overfitting by randomly deactivating neurons during training. The final classification is performed using fully connected layers, including one with 128 neurons and ReLU activation, followed by a softmax output layer with three neurons corresponding to the burn categories (superficial, dermal, full-thickness).

### RGB to LUV conversion and Gaussian filtering

The conversion from RGB to LUV is essential for several reasons. The LUV color model offers a more intuitive and perceptually meaningful representation of colors compared to the device-dependent RGB model. The L* coordinate signifies luminance, while U* and V* coordinates characterize colors along green-to-red and blue-to-yellow spectra. Gaussian filtering of the L*, U*, and V* images using low-pass filters reduces high-frequency noise and enhances color information. This preprocessing step improves the robustness of subsequent analyses by emphasizing essential color features, thus contributing to the overall quality and reliability of color-based analysis.

Gaussian filtering is a widely used technique in image processing for noise reduction and image smoothing. This is particularly important in the context of burn image analysis, where high-frequency noise can obscure critical tissue features. By reducing such noise, Gaussian filtering enhances the visibility of structural details, facilitating downstream analysis tasks such as segmentation and classification. The Gaussian filter operates by applying a two-dimensional convolution between the input image $I(x, y)$ and a Gaussian kernel $G(x, y)$. The Gaussian kernel is mathematically defined as:

2 $$\begin{aligned} G(x, y) = \frac{1}{2\pi \sigma ^2} e^{-\frac{x^2 + y^2}{2\sigma ^2}}, \end{aligned}$$

where $x$ and $y$ represent the spatial coordinates relative to the center of the kernel, and $\sigma$ denotes the standard deviation of the Gaussian distribution. The parameter $\sigma$ controls the degree of smoothing, with larger values producing greater blurring and smaller values preserving finer details. The filtered image $I'(x, y)$ is computed as:

3 $$\begin{aligned} I'(x, y) = \sum _{u,v} I(x-u, y-v) \cdot G(u, v), \end{aligned}$$

where $(u, v)$ are the coordinates within the Gaussian kernel. This convolution operation effectively averages pixel intensities within a local neighborhood, weighted by the Gaussian function, thereby attenuating sharp intensity variations. The effect of Gaussian filtering on an image can be visually observed by comparing the original and filtered versions. In the original image, high-frequency noise and abrupt intensity transitions are apparent, which can obscure important tissue structures. After applying Gaussian filtering, the image appears smoother, with reduced noise and enhanced clarity of tissue features. This preprocessing step is critical for improving the performance of subsequent tasks, such as texture analysis and segmentation.

For this study, a Gaussian kernel of size $5 \times 5$ and a standard deviation $\sigma = 1.5$ were employed, as these parameters were experimentally determined to balance noise reduction with feature preservation. Gaussian filtering, therefore, serves as a foundational step in the image preprocessing pipeline, ensuring that the input images are optimized for accurate and reliable burn injury analysis.

### Dynamic contrast enhancement and kinetic models

Dynamic contrast enhancement (DCE) using the LUV model, guided by kinetic models like the Tofts model, extends beyond burn profile analysis to various medical fields. This approach provides a noninvasive means to assess tissue perfusion and permeability, which are essential for understanding pathophysiology. Integrating DCE into clinical practice addresses the need for accurate burn profile estimation and monitoring without invasive procedures, empowering healthcare professionals with a powerful tool to evaluate treatment responses. As the demand for precise, personalized medicine continues to rise, DCE, guided by advanced models, is set to play an increasingly vital role in modern healthcare.

In this work, the assessment of burn injuries using Dynamic Contrast Enhancement (DCE) imaging is guided by kinetic models, particularly the Tofts model. These models provide a quantitative framework for analyzing the uptake and distribution of contrast agents, offering valuable insights into tissue perfusion and vascular properties. The Tofts model is a pharmacokinetic framework commonly employed in medical imaging to describe the movement of contrast agents between the blood plasma and the extracellular extravascular space (EES). The model is mathematically represented as:

4 $$\begin{aligned} C_t(t) = K^{\text {trans}} \int _0^t C_p(\tau ) e^{-\frac{K^{\text {trans}}}{v_e} (t-\tau )} d\tau , \end{aligned}$$

where:

- $C_t(t)$: Concentration of the contrast agent in the tissue at time $t$,
- $C_p(t)$: Concentration of the contrast agent in the plasma,
- $K^{\text {trans}}$: Transfer constant, describing the rate of contrast agent movement from plasma to EES,
- $v_e$: Volume fraction of the EES.

The Tofts model is instrumental in burn imaging as it provides a means to quantify the kinetic patterns of contrast agents in burned versus unburned tissue. Regions with compromised vasculature, such as full-thickness burns, exhibit distinct kinetic behaviors when compared to superficial burns or healthy tissue. By analyzing these differences, the model helps to quantify perfusion deficits that are closely correlated with the severity of tissue damage.

Using this model, kinetic parameters such as $K^{\text {trans}}$ and $v_e$ are derived and integrated into the computational pipeline. These parameters enhance the accuracy of segmentation and classification algorithms, enabling precise estimation of burn depth and Total Body Surface Area (TBSA). The Tofts model thus plays a pivotal role in improving the reliability of burn injury assessment through DCE imaging.

### Algorithm for adaptive complex independent components analysis and Fuzzy C-Means (FCM) clustering

The Pixel Difference Function (PDF) is a computational metric used to quantify variations in pixel intensity values within a defined region of an image. This function is particularly relevant in burn image analysis, where intensity gradients and spatial distributions can indicate transitions between different burn regions. By analyzing these variations, the PDF helps identify boundaries and highlight regions with significant intensity fluctuations, which are often associated with heterogeneous tissue damage. The PDF for a given region is mathematically defined as:

5 $$\begin{aligned} \text {PDF}(i, j) = |I(i, j) - I(i+1, j+1)|, \end{aligned}$$

where:

- $I(i, j)$: Intensity value of the pixel at position $(i, j)$,
- $I(i+1, j+1)$: Intensity value of the neighboring pixel in a predefined direction.

This formulation calculates the absolute difference between adjacent pixel intensities. By doing so, the PDF effectively captures local variations within the image, which correspond to edges or transitions between regions of varying burn severity. For instance, regions with sharp intensity differences may indicate the boundary between superficial and full-thickness burns.

The application of the PDF within this study is twofold. First, it aids in detecting edges and boundaries between burn regions, enabling accurate segmentation. Second, it highlights regions with significant intensity fluctuations, which may correspond to heterogeneous or progressive tissue damage. These insights are crucial for burn classification and depth estimation, as they provide a quantitative basis for distinguishing between different burn severities. By incorporating the PDF into the computational framework, this study ensures enhanced precision in burn region analysis, thereby supporting reliable segmentation and improving diagnostic accuracy.

Accurate assessment of agent contrast concentration and pixel clustering is pivotal in medical image analysis, particularly for dynamic contrast-enhanced burn samples. This algorithm presents a systematic procedure for calculating agent contrast concentration and performing pixel clustering using FCM, a robust technique for image data segmentation. The algorithm 1 initiates by converting the input image to a double data type and initializing essential variables such as *ncluster*, *g*, *PDF*, *gbar*, and *Intra contrast agent (ICA)*. It calculates region averages, computes the pixel difference function (PDF) to understand contrast distribution, and determines the average cluster center *gbar*. The core of the algorithm involves FCM clustering, which segments the image into clusters based on pixel similarities and assigns cluster levels. The leveled image visually represents agent contrast concentration distribution, serving as a valuable resource for medical image analysis.

This algorithm 1 provides a systematic approach to compute agent contrast concentration and perform pixel clustering through FCM clustering. The detailed steps, including region average calculations, PDF computation, and FCM clustering, ensure a comprehensive analysis of the image data. The leveled image illustrates the distribution of agent contrast concentration, enhancing the utility of medical image analysis for diverse diagnostic and research applications. Algorithm 1 describes a computational procedure known as Adaptive Complex Independent Components Analysis and FCM Clustering. This algorithm is particularly useful for analyzing images, as it calculates average contrast values and pixel differences to enhance image clarity and facilitate further analysis. The algorithm begins by taking as input an image $I$ and the desired number of clusters $n_{\text {cluster}}$. It then proceeds through several key steps:

**Algorithm 1:**
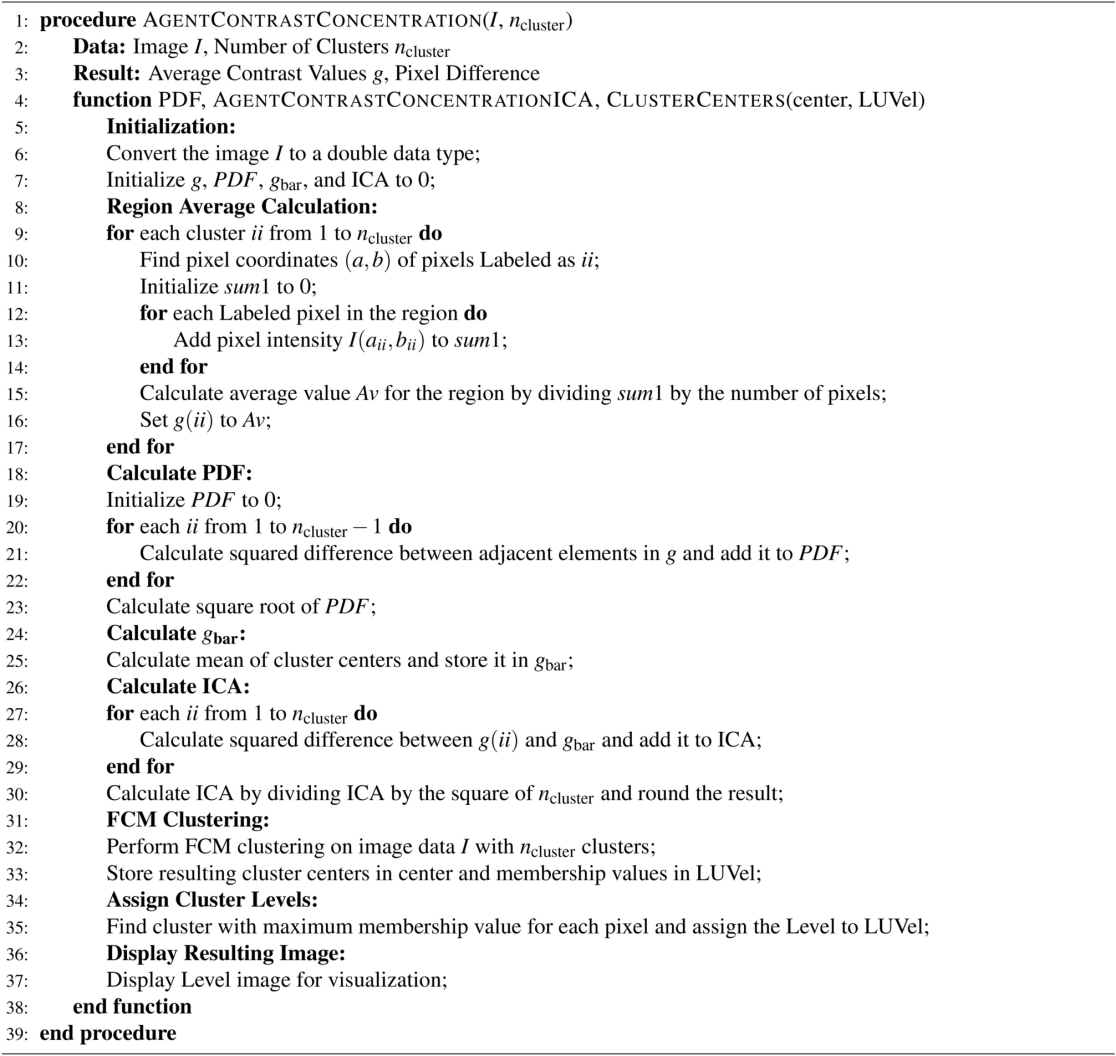
Adaptive Complex Independent Components Analysis and Fuzzy C-Means (FCM) Clustering

1. Initialization: The image is converted to a double data type, and various variables such as average contrast values ($g$), PDF (Pixel Difference Function), cluster centers ($\text {center}$), and Agent Contrast Concentration ($\text {ICA}$) are initialized to zero.
2. Region Average Calculation: For each cluster, the algorithm identifies the pixels labeled with that cluster and calculates the average pixel intensity for that region. These average values ($g(ii)$) represent the contrast concentration within each region.
3. Calculation of PDF: The algorithm calculates the Pixel Difference Function (PDF) by computing the squared differences between adjacent average contrast values ($g$). This step provides insight into the overall contrast variation across neighboring regions.
4. Calculation of $g_{\text {bar}}$: The mean of the cluster centers is computed and stored as $g_{\text {bar}}$, which serves as a reference point for assessing the deviation of each region’s contrast concentration.
5. Calculation of ICA: Agent Contrast Concentration (ICA) is computed by measuring the squared differences between each region’s contrast concentration ($g(ii)$) and the mean contrast concentration ($g_{\text {bar}}$). This step quantifies the variability of contrast concentration across different regions.
6. FCM Clustering: Fuzzy C-Means (FCM) clustering is performed on the image data to partition the image into $n_{\text {cluster}}$ clusters based on the calculated contrast concentrations. This clustering step helps identify distinct regions with similar contrast characteristics.
7. Assigning Cluster Levels: For each pixel in the image, the algorithm determines the cluster with the maximum membership value (indicating the highest similarity) and assigns the corresponding cluster level (LUVel) to that pixel. This step facilitates the segmentation of the image into different contrast levels.
8. Displaying Resulting Image: Finally, the resulting image, segmented into different contrast levels, is displayed for visualization, providing insights into the contrast distribution and structure within the image.

**Algorithm 2:**
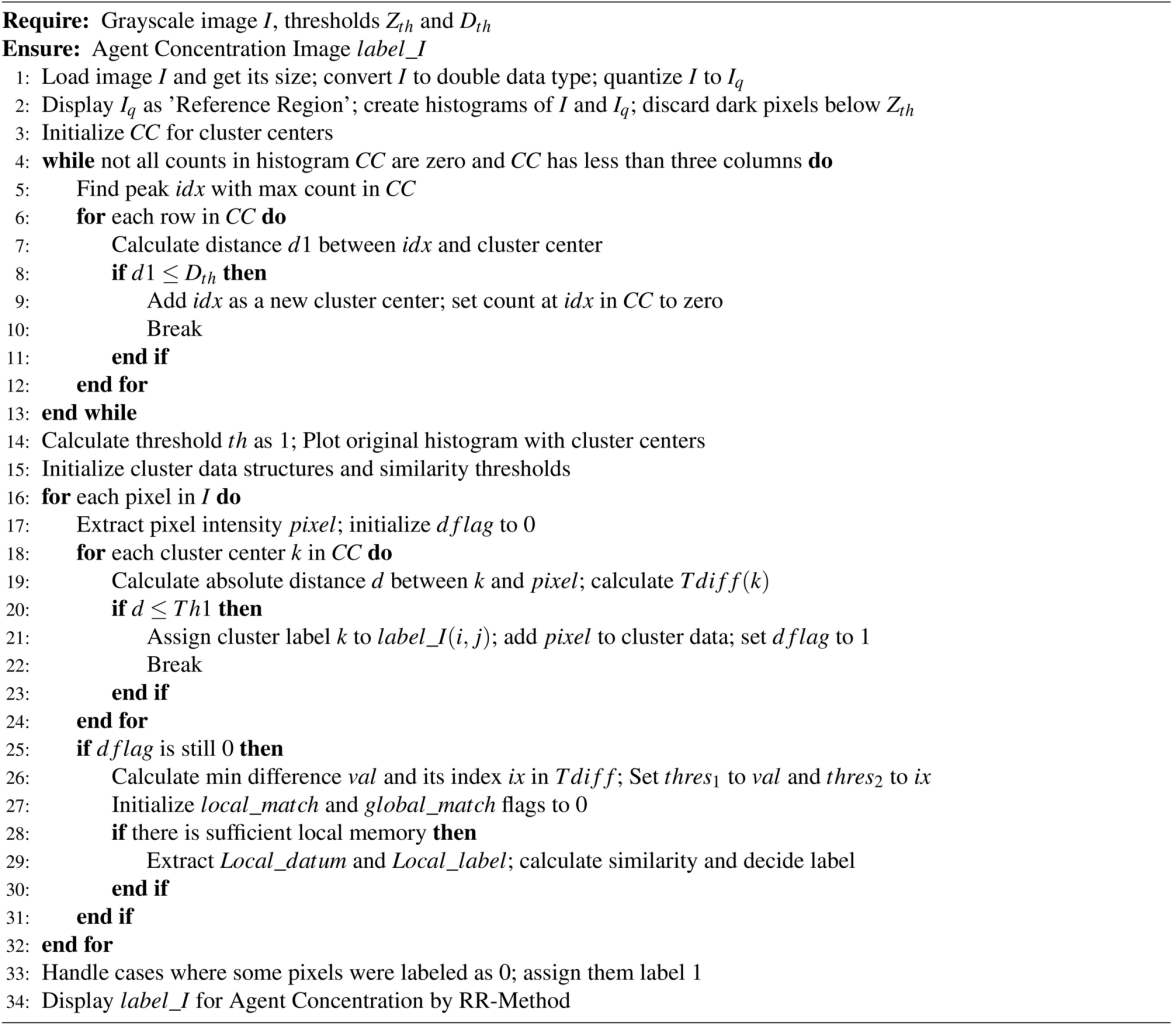
Agent Concentration by RR-Method

Algorithm 2, titled “Agent Concentration by RR-Method,” outlines a procedure for processing grayscale images to produce an Agent Concentration Image. It involves several key steps aimed at identifying clusters of pixel intensities and assigning labels to pixels based on their similarity to these clusters. Initially, the algorithm loads a grayscale image *I* and quantizes it to $I_q$, discarding dark pixels below a specified threshold $Z_{th}$. It then constructs histograms of both the original and quantized images and identifies peaks in the histogram as potential cluster centers. These centers are iteratively added until certain conditions are met, ensuring an adequate representation of pixel-intensity clusters.

Subsequently, the algorithm calculates a threshold *th* based on the histogram and plots the original histogram with the identified cluster centers for visualization. It initializes cluster data structures and similarity thresholds before proceeding to label each pixel in the image. For each pixel, the algorithm compares its intensity to each cluster center’s intensity and assigns it a label corresponding to the closest center if it meets certain criteria. If no suitable cluster center is found, the algorithm calculates a minimum difference between the pixel intensity and the cluster centers, determining a new threshold and handling cases where some pixels remain unlabeled.

Finally, the algorithm ensures that all pixels are labeled, assigning label 1 to any remaining unlabeled pixels. The resulting image, labeled as $label\_I$, represents the concentration of agents in the original image, providing insights into the distribution of pixel intensities and facilitating further analysis. Algorithm 2 offers a systematic approach to segmenting grayscale images based on intensity clusters, allowing for the generation of an Agent Concentration Image using the RR-Method.

### Feature extraction

Feature extraction constitutes a cornerstone of our research, essential for deriving meaningful insights from complex image data. Our approach hinges on harnessing the Grey-Level Co-occurrence Matrix (GLCM) technique, meticulously applied to two distinct sources: decomposed luminance and color components. Through this method, we extract fundamental texture features, specifically contrast and correlation, crucial for characterizing the intricate details within the imagery.

The GLCM is a widely used texture analysis method in image processing for characterizing spatial relationships between pixel intensities. In this study, GLCM is employed to extract texture features that are critical for differentiating burn regions and depths, providing insights into the structural heterogeneity of tissue. A GLCM is constructed by considering pairs of pixels separated by a defined distance $d$ and orientation $\theta$ (e.g., $0^\circ$, $45^\circ$, $90^\circ$, $135^\circ$). The matrix element $P(i, j)$ represents the normalized frequency of two pixels with intensities $i$ and $j$ occurring at the specified spatial relationship:

6 $$\begin{aligned} P(i, j) = \frac{\text {Number of pixel pairs with intensities } i \text { and } j}{\text {Total number of pixel pairs}}. \end{aligned}$$

From the GLCM, key texture features are derived to quantify the statistical properties of the image.

**Contrast** measures the intensity variation between neighboring pixels:

7 $$\begin{aligned} \text {Contrast} = \sum _{i,j} (i - j)^2 P(i, j). \end{aligned}$$

Higher contrast values indicate sharper intensity changes, highlighting edges and boundaries.

**Correlation** quantifies the linear dependency of pixel intensities:

8 $$\begin{aligned} \text {Correlation} = \sum _{i,j} \frac{(i - \mu _i)(j - \mu _j) P(i, j)}{\sigma _i \sigma _j}, \end{aligned}$$

where $\mu _i, \mu _j$ are the means and $\sigma _i, \sigma _j$ are the standard deviations of intensity values in the row and column directions, respectively.

Higher homogeneity values indicate more uniform textures. GLCM-derived features have been extensively validated in medical imaging applications. Studies, such as Bakheet et al.^60^ (2021) and Yuan et al.^61^ (2024), have demonstrated that these features correlate strongly with clinical observations of tissue heterogeneity and damage severity. In this study, the GLCM technique was validated using a dataset of annotated burn images. Extracted texture features were compared against expert evaluations of burn depth and texture, and statistical metrics such as accuracy and F1-score confirmed the utility of GLCM features in classifying burn severity.

The extracted features enable the classification of burn regions into superficial, dermal, and full-thickness burns by capturing textural differences. This process significantly enhances the accuracy of segmentation and depth estimation, facilitating reliable diagnostic conclusions.

To ensure thorough texture analysis across various orientations, we employ an $8 \times 8$ mask with offsets at $0^\circ$, $45^\circ$, $90^\circ$, and $135^\circ$ during the calculation process. This meticulous approach enables us to capture nuanced texture variations comprehensively, enhancing the accuracy of our feature extraction.

Furthermore, we augment our feature extraction process by incorporating the means of luminance-color images. This integration allows us to capture additional texture nuances, thus enriching the representation of features extracted from the images. As a result, we obtain feature vectors consisting of 10 values, with each luminance-color source contributing 5 values. These feature vectors serve as crucial input data for subsequent cluster analysis.

By leveraging these rich and informative feature vectors, our cluster analysis can effectively identify and delineate burn regions within the images with heightened accuracy and reliability. This meticulous feature extraction process forms the bedrock of our methodology, empowering us to extract actionable insights from complex image datasets.

The calculation of GLCM (Gray-Level Co-occurrence Matrix) contrast and correlation involves several steps:

This Algorithm 3 begins by extracting the L value from the input image *I* if color space information *LUV* is provided. Then, it applies the GLCM method to compute two GLCMs, one for *L* and another for $L^2$. Subsequently, it calculates the contrast and correlation from the GLCMs using defined formulas. Finally, it displays the computed contrast and correlation values or creates a graph to visualize them, completing the process.

The provided Algorithm 3 outlines a systematic procedure for computing two essential metrics, contrast, and correlation, from Gray-Level Co-occurrence Matrices (GLCMs), which capture relationships between pixel intensity values in images. Initially, the Algorithm 3 receives input in the form of an image $I$ and optionally color space information $LUV$, extracting the luminance component $L$ from $I$ if $LUV$ is provided. Subsequently, two GLCMs are computed using the GLCM method, one for the luminance component $L$ and another for its squared value $L^2$.

Next, the Algorithm 3 calculates contrast, a measure of local intensity variation, from the GLCM. It iterates through each pair of gray levels in the GLCM, computing the squared difference between their intensities multiplied by the corresponding GLCM value, and then sums up these products to obtain the overall contrast value. Following this, the Algorithm 3 computes correlation, which indicates the linear relationship between pixel intensities. It calculates mean and standard deviation values for row and column sums of the GLCM, representing the mean intensity and intensity variation in different directions, and then iterates through each pair of gray levels in the GLCM, calculating the correlation contribution for each pair and summing them up to obtain the overall correlation value.

Finally, the computed contrast and correlation values can be displayed directly or visualized through graphs for better interpretation. This step aids in understanding the textural characteristics of the image, providing insights into its local intensity variations and linear intensity relationships. The Algorithm 3 offers a structured approach to compute GLCM contrast and correlation, facilitating texture analysis tasks in image processing.

**Algorithm 3:**
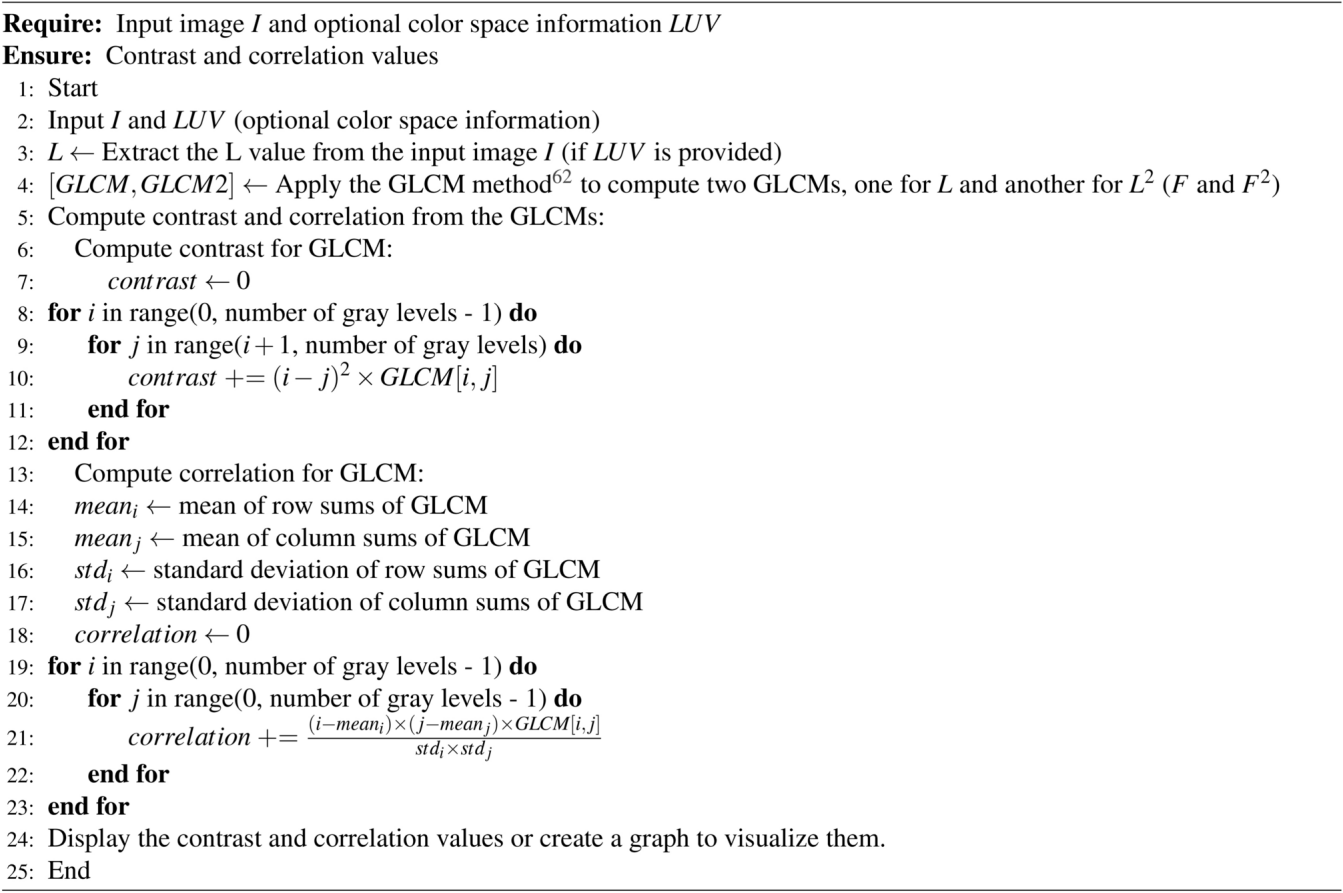
Calculation of GLCM Contrast and Correlation

Burn profile analysis involves the examination of tissue samples at a microscopic level, primarily focusing on texture analysis to identify distinct characteristics. The pseudocode provided outlines a systematic framework for extracting valuable texture information from burn images.

The process begins by acquiring the burn profile image (I) as input. While an optional step for extracting the L channel in color images (LUV) is included, it is noteworthy that most burn profile images are grayscale. The core of the analysis lies in computing Gray-Level Co-occurrence Matrices (GLCMs) for the grayscale histopathology image. GLCMs capture the spatial relationship of pixel values at various distances and angles within the image. In this method, two GLCMs are calculated: one directly from the grayscale image (L) and another from the squared values of pixel intensities (L squared). These matrices are essential for characterizing the texture of the burn profile image.

From the GLCMs, two crucial texture features are computed: contrast and correlation. Contrast measures the local intensity variations within the image, highlighting regions with significant differences in pixel values. In burn profile images, high contrast values can indicate areas with diverse textures or structural variations. Correlation quantifies the linear relationship between pixel values. High correlation values suggest that pixel intensities tend to vary together, revealing structured patterns or textures in the image.

The computed contrast and correlation values can be displayed or used to generate graphs. Such visualizations aid in identifying regions of interest within burn profile images, potentially pinpointing areas with specific texture characteristics. This is valuable for diagnosing diseases, detecting anomalies, or conducting medical research involving burn profile images.

In the proposed methodology, the process starts by taking a query image as input. The image is then converted from RGB to LUV color space, a crucial step for standardizing the color representation and making it more perceptually uniform. From the LUV-converted image, the GLCM contrast and correlation are calculated. These features are then used to train a model with all database images. After training, the GLCM features are extracted from the database images. This trained model is used to classify and analyze new burn profile images.

**Fig. 5:**
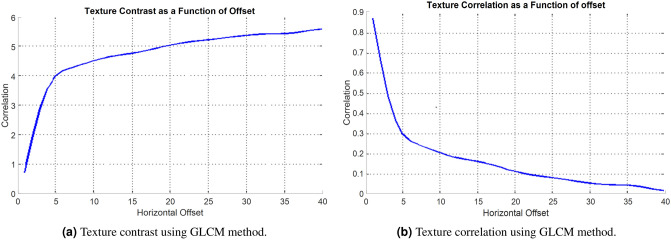
Comparison of texture contrast and texture correlation using the GLCM method.

Figure 5a showcases the use of GLCM methods for texture analysis in burn profile images. The plot serves as a visual representation, providing insights into how texture contrast values change with varying horizontal offsets. The horizontal (x) axis, ranging from 0 to 40, represents the “horizontal offset,” indicating the spatial distance over which pixel pairs are evaluated. This parameter reflects the texture scale or granularity under investigation. The vertical (y) axis, with a range from 0 to 6, represents the “contrast” values. Higher contrast values signify more pronounced textural differences within the image. The plot visually illustrates how contrast values change as the horizontal offset varies, providing insights into the texture contrast within burn profile images.

Figure 5b presents the texture correlation using the GLCM method. The horizontal (x) axis, spanning from 0 to 40, represents the “horizontal offset,” a key parameter in GLCM analysis. This offset signifies the spatial distance over which pixel pairs are evaluated, reflecting the scale or granularity of the texture under scrutiny. The vertical (y) axis, ranging from 0 to 0.9, represents “correlation” values. In GLCM-based texture analysis, correlation quantifies the degree of relationship between pixel pairs at various distances. Values closer to 1 indicate a strong positive correlation, suggesting more uniform or repetitive texture patterns in the image. This plot visually illustrates how correlation values change as the horizontal offset varies. It offers insights into whether the texture is consistent or varies with changes in distance, which can be invaluable for distinguishing different tissue structures and textural characteristics within the burn profile.

These analyses, encompassing both contrast and correlation features derived from GLCMs, provide a comprehensive understanding of the textural properties of burn profile images. This understanding supports improved diagnosis and research in the field, enhancing the accuracy and depth of medical assessments and contributing to better patient outcomes.

### Model construction and measurement of accuracy

#### Image analysis tool for burn assessment

The image analysis tool presented in this study integrates a range of advanced computational techniques to analyze burn injuries comprehensively. It employs Adaptive Complex Independent Component Analysis (ACICA) and Reference Region (RR) methods to segment input images into distinct regions corresponding to healthy skin, superficial burns, partial-thickness burns, and full-thickness burns. These segmentation methods rely on contrast and texture variations observed in the image, enabling accurate delineation of burn regions.

Following segmentation, the tool applies texture analysis techniques, such as the Grey Level Co-occurrence Matrix (GLCM), to extract critical features, including contrast and correlation. These features are mapped to burn depth categories using a supervised machine learning model, which has been trained to classify burns into three primary categories: superficial, partial-thickness, and full-thickness burns. This classification is based on subtle textural and contrast variations that are indicative of burn severity.

The tool also estimates the Total Body Surface Area (TBSA) of burns for individual samples by considering the entire sample as 100%. For each sample, the tool calculates the percentage of the image affected by different burn depths. While this method deviates from the traditional clinical definition of TBSA, which estimates the percentage of the total body surface affected in a patient, it provides granular insights into the burn distribution within the analyzed sample. These insights are valuable for understanding the severity and distribution of burns at a localized level.

By integrating segmentation, texture analysis, and TBSA estimation, the tool offers a comprehensive solution for analyzing burn injuries. Its potential applications extend to clinical diagnostics^63^, treatment planning, and patient monitoring, enabling more precise and data-driven approaches to burn care.

#### Convolutional Neural Network (CNN)

The proposed framework utilizes a CNN optimized for the classification of burn types, such as superficial, partial-thickness, and full-thickness burns. The architecture is initiated with an input layer that processes burn sample images standardized to dimensions of 224 $\times$ 224 $\times$ 3, ensuring consistency in input data. Three convolutional layers are used to execute feature extraction, each having the kernel size 33 $\times$ 3, followed by a ReLU activation for enhancing nonlinear feature learning of the network. The number of convolutional layers increases from 32 filters in the first layer, followed by 64 in the second, and 128 in the third, enabling progressive captures of higher complexities of the image features. After each convolutional layer, a max-pooling layer is applied with a pool size of 22$\times$2 to downsample the feature maps, which reduces the spatial dimensions and computational complexity while maintaining the important features.

To avoid overfitting and ensure generalization, dropout layers with a dropout rate of 0.25 have been added after each of the pooling layers. Finally, the feature map is flattened to a one-dimensional vector via a flattened layer that acts as the input for the fully connected layers. The architecture consists of two dense layers: the first one contains 128 neurons and ReLU activation, while the output layer has three neurons, corresponding to the three categories of burns. The output layer will use a softmax activation function in order to provide probability distributions across the three categories of burns. This CNN architecture, which combined feature extraction, regularization, and classification layers, was rigorously optimized through cross-validation, resulting in robust performance in the classification of burn severity. The framework effectively supports clinical decision-making by providing accurate and reliable burn type predictions. .

The output of a convolutional layer is calculated using the convolution operation, represented as:

9 $$\begin{aligned} Y[i, j] = \sum _{m}\sum _{n} X[i+m, j+n] \cdot W[m, n] + b \end{aligned}$$

where:

- $Y[i, j]$ is the result of the feature map at point $(i, j)$.
- $X[i+m, j+n]$ denotes the input feature map at the spatial location $(i+m, j+n)$.
- $W[m, n]$ represents the filter or convolution kernel, a small matrix of weights learned during the training phase to identify specific features.
- $b$ is the bias term.

In this study^64^, the categorical cross-entropy loss function was utilized to train the deep neural network for burn classification. This loss function is particularly well-suited for multi-class classification tasks as it measures the divergence between the predicted probability distribution of the model and the true class labels. By minimizing this loss, the model learns to improve its predictions for the correct classes while reducing the likelihood of incorrect classifications.

The categorical cross-entropy loss is mathematically defined as:

10 $$\begin{aligned} \mathscr {L} = -\frac{1}{N} \sum _{i=1}^{N} \sum _{j=1}^{C} y_{ij} \log (p_{ij}), \end{aligned}$$

where $N$ represents the total number of samples, $C$ is the total number of classes (e.g., superficial, dermal, and full-thickness burns), $y_{ij}$ is a binary indicator (0 or 1) denoting whether sample $i$ belongs to class $j$, and $p_{ij}$ is the predicted probability for class $j$ of sample $i$.

Equation 10 penalizes the model heavily for larger discrepancies between the predicted probabilities $p_{ij}$ and the true labels $y_{ij}$. For instance, if the model assigns a low probability to the correct class ($y_{ij} = 1$), the term $\log (p_{ij})$ becomes highly negative, increasing the overall loss. Conversely, when the model predicts the correct class with high confidence ($p_{ij} \approx 1$), the contribution to the loss is minimal. This mechanism ensures that the model improves its confidence in the correct class while minimizing probabilities assigned to incorrect classes.

#### Training process and model optimization

The dataset used in this study consists of 90 burn sample images acquired from clinical settings. Each image was manually labeled by expert clinicians into three burn depth categories: superficial burns, partial-thickness burns, and full-thickness burns. This manual labeling process ensured accuracy and consistency, forming a reliable foundation for training the machine learning model.

To effectively train and evaluate the model, the dataset was divided into training, validation, and test sets using a stratified approach to maintain an equal distribution of burn depth categories across all subsets. The training set consisted of 70% of the dataset (63 images), the validation set comprised 15% (14 images), and the remaining 15% (13 images) formed the test set. This division ensured that the model was trained on a representative subset of the data while retaining sufficient samples for validation and testing.

The model was trained for 50 epochs, where each epoch represented a complete pass through the training dataset. To prevent overfitting and ensure that the model did not learn spurious patterns based on image order, the images were randomly shuffled at the beginning of each epoch. Additionally, data augmentation techniques were applied to artificially expand the dataset and improve the model’s generalization ability. These augmentation techniques included rotation ($\pm 15^\circ$), horizontal and vertical flipping, brightness adjustments ($\pm 20\%$), and zooming ($\pm 10\%$). These transformations ensured variability in the training data while preserving essential image features.

A five-fold cross-validation approach was employed to further validate the model. In each fold, the dataset was split into training (80%) and testing (20%) subsets, and the process was repeated five times, with a different subset reserved for testing in each iteration. The final model performance metrics were averaged across all five folds to ensure robustness and reliability, mitigating the effects of potential data imbalances.

The classification task involved categorizing each burn sample image into one of three burn depth categories: superficial burns, partial-thickness burns, and full-thickness burns. The classification relied on texture features extracted during preprocessing, enabling the model to capture subtle variations indicative of burn depth.

The proposed framework employed a Convolutional Neural Network (CNN) optimized for burn type classification. The architecture began with an input layer that processed standardized burn sample images with dimensions of $224 \times 224 \times 3$, ensuring consistency in input data. Feature extraction was performed through three convolutional layers, each employing a $3 \times 3$ kernel size and followed by a Rectified Linear Unit (ReLU) activation function to enhance non-linear feature learning. The number of filters progressively increased across the layers, starting with 32 in the first layer, 64 in the second, and 128 in the third. This hierarchical structure enabled the network to capture increasingly complex features relevant to burn depth classification.

Max-pooling layers with a $2 \times 2$ pool size were applied after each convolutional layer to downsample the feature maps, reducing spatial dimensions and computational complexity while preserving essential features. To further prevent overfitting and improve generalization, dropout layers with a dropout rate of 0.25 were incorporated after each pooling layer.

The final feature map was flattened into a one-dimensional vector through a flatten layer, which served as input to the fully connected layers. The architecture included two dense layers: the first dense layer consisted of 128 neurons with ReLU activation, while the output layer contained three neurons corresponding to the three burn depth categories. The output layer employed a softmax activation function to generate probability distributions across the categories, enabling precise classification.

This CNN architecture, rigorously optimized through cross-validation, demonstrated robust performance in classifying burn severities. By leveraging both data augmentation and systematic validation strategies, the framework effectively supports clinical decision-making by providing precise and reliable predictions of burn types.

#### Recurrent Neural Network (RNN)

A Recurrent Neural Network (RNN) is a neural network architecture designed to handle sequential data, such as text or time-series data. Unlike feedforward neural networks that process inputs independently, RNNs capture temporal dependencies by maintaining a hidden state that reflects previous information through recurrent connections. At each time step, the hidden state is updated based on the current input and the previous hidden state, forming a recurrent loop. This allows the network to remember past inputs and identify temporal relationships in the data. However, RNNs face challenges such as the vanishing gradient problem, particularly with long sequences, where gradients exponentially decay as they propagate backward in time, making it difficult to learn long-term dependencies.

The hidden state of an RNN at time step $t$ is calculated using the following equation:

11 $$\begin{aligned} h_t = \sigma ( W_{hh} \cdot h_{t-1} + W_{xh} \cdot x_t + b_h ) \end{aligned}$$

where:

- $W_{hh}$ and $W_{xh}$ are weight matrices.
- $b_h$ is the bias term.
- $\sigma$ is the activation function.
- $h_t$ is the hidden state at time $t$.

#### Feedforward Neural Network (FNN)

A Feedforward Neural Network (FNN), also known as a Multilayer Perceptron (MLP), is the simplest type of neural network architecture where data flows in one direction, from the input layer to the output layer, through one or more hidden layers. Each neuron in a hidden layer produces an output by applying an activation function to the weighted sum of its inputs from the previous layer. This non-linearity allows the network to learn complex relationships and patterns in the data. FNNs are often used for tasks such as regression and classification, where the input-output mapping is straightforward and does not involve spatial or sequential dependencies. Despite their simplicity, FNNs have proven useful in a wide range of applications due to their ability to model complex data. The output of a neuron in an FNN is given by:

12 $$\begin{aligned} z = \sum _{i} w_i \cdot x_i + b \end{aligned}$$

where:

- $w_i$ are the weights.
- $x_i$ are the input values.
- $b$ is the bias term.
- $z$ is the weighted sum.

The neuron’s output is then obtained by passing this weighted sum through a nonlinear activation function, typically denoted as $a = f(z)$, where $f$ is the activation function.

### Multi-Modal assessment framework

The proposed Multi-Modal Assessment framework incorporates a range of complementary techniques to ensure accurate and comprehensive evaluation of burn injuries. While Dynamic Contrast Enhancement (DCE) is utilized as one of the methods to highlight tissue characteristics, the framework is designed to operate effectively without relying solely on DCE-derived metrics.

Enhancing the luminance ($L$) channel of the image enables the identification of subtle intensity differences within the burn area, such as boundaries between healthy and affected skin or varying degrees of tissue damage. This approach provides critical visual insights into burn severity without requiring contrast enhancement.

The framework also employs Grey Level Co-occurrence Matrix (GLCM) texture analysis to extract essential features, including contrast and correlation, which are indicative of tissue properties and burn depth. These texture-based metrics are calculated directly from the raw image data, offering valuable insights independent of any contrast-enhanced imaging.

For automated classification, deep learning models such as Convolutional Neural Networks (CNNs) are utilized to analyze raw image features and texture data. The CNNs are trained to differentiate burn types-superficial, partial-thickness, and full-thickness burns-based on intrinsic image properties. This model-driven approach significantly improves diagnostic accuracy and reduces dependence on DCE-derived features.

Segmentation techniques, such as Adaptive Complex Independent Component Analysis (ACICA) and Reference Region (RR) methods, are integrated into the framework to identify distinct regions and estimate burn depth. These methods are designed to operate effectively across a variety of imaging modalities, ensuring adaptability and robustness in diverse clinical scenarios.

This integrative approach ensures that burn assessment is not dependent on DCE alone. By leveraging a combination of luminance enhancement, texture analysis, machine learning, and segmentation techniques, the framework delivers accurate, reliable, and clinically applicable results for burn injury evaluation.

## Results

This section presents an extensive exploration of the results and their interpretation, emphasizing the importance of our study’s findings. We systematically showcase, scrutinize, and contextualize the data obtained from meticulous experiments, surveys, and analyses, aiming to provide a comprehensive understanding of the ramifications and significance of our research within the broader field.

**Fig. 6:**
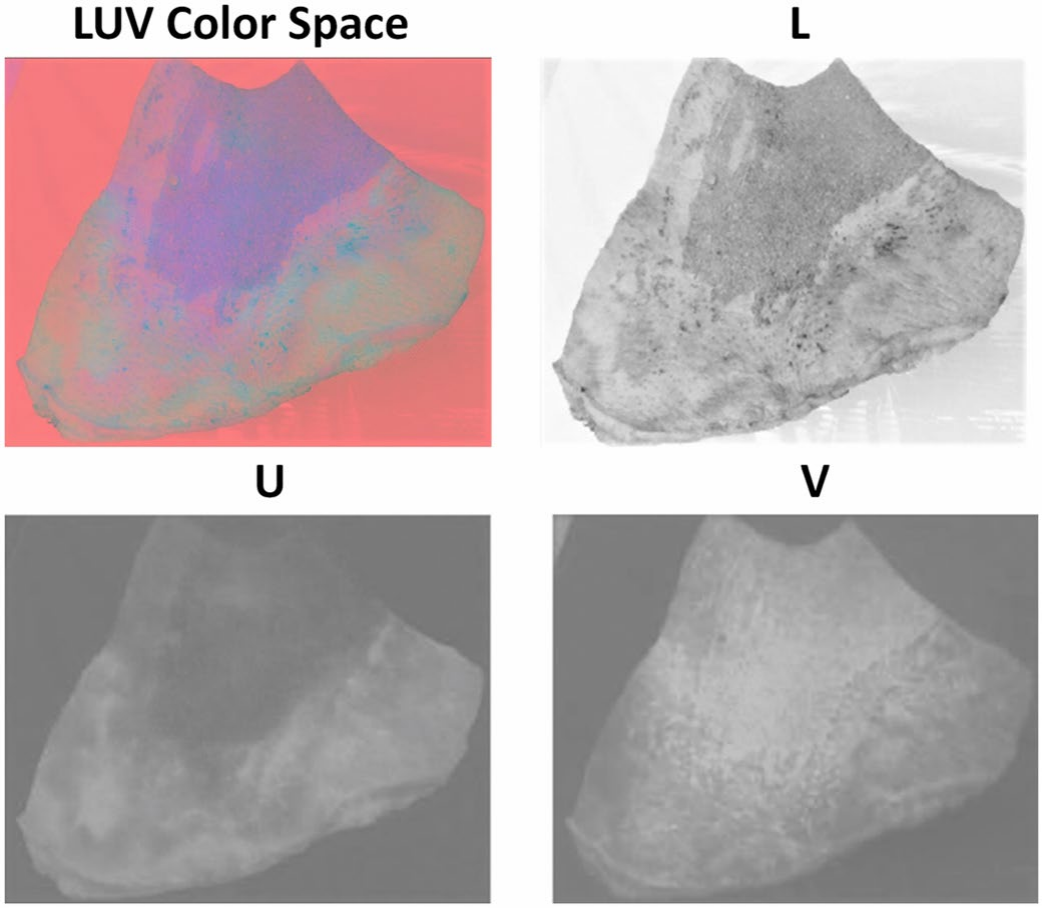
LUV Image: The reference image used as a basis for burn degree analysis.

We begin with the RGB to LUV converted input image shown in Figure 6, selected from our extensive database. The LUV color model offers a more intuitive and perceptually meaningful representation of colors compared to the device-dependent RGB model, serving as a crucial component for burn profile estimation. This input image acts as the initial point of reference for the system’s subsequent actions. During this step, the system’s algorithms and processes are primed to read and analyze the selected sample image, laying the foundation for comprehensive analysis and comparison. This process ultimately leads to the retrieval of relevant medical images that closely match the query image’s characteristics and features.

Statistical parameters are analyzed and tabulated in Table 2 by applying dynamic contrast enhancement for burn profile estimation. These parameters include mean, standard deviation, entropy, skewness, and kurtosis, offering valuable insights into the characteristics of different tissue layers within burn profiles. This detailed analysis is essential for accurate medical assessment and treatment planning.

**Table 2:** Statistical Analysis of Query Image and DCE Preprocessed Image.

**Statistical parameter**	**Query image**	**DCE-PP image**
Mean	56.65	76.87
Standard Deviation	18.24	15.35
Entropy	5.68	6.25
Skewness	2.05	2.56
Kurtosis	125.69	169.35

The statistical parameters collectively reflect the diversity in contrast behavior within burn profile samples. The fluctuation in mean, standard deviation, entropy, skewness, and kurtosis values may indicate variations in contrast behavior and tissue attributes. A higher mean in trained images could imply regions with increased contrast uptake, potentially signifying burn areas. The greater standard deviation in images might indicate more dynamic contrast behavior relevant for detecting aggressive burn progression. Elevated entropy, positive skewness, and increased kurtosis collectively suggest intricate and dynamic contrast kinetics, potentially linked to aggressive burn development. While each parameter offers insights, no single “best” parameter exists for burn profile estimation. A comprehensive approach that considers multiple parameters and their interactions proves more effective. Integrating these statistical measures with clinical and imaging data can significantly enhance the accuracy of burn profile characterization and estimation.

### Comparative analysis of feature extraction methods

The impact of our ACICA method is significant, especially in contrast to the conventional Principal Component Analysis (PCA) and ICA methods. The ACICA and RR methods demonstrate superior performance across various metrics, establishing them as valuable advancements in burn wound analysis. Our comparative analysis of feature extraction methods indicates that the ACICA approach provides more accurate and reliable results for burn depth classification.

**Fig. 7:**
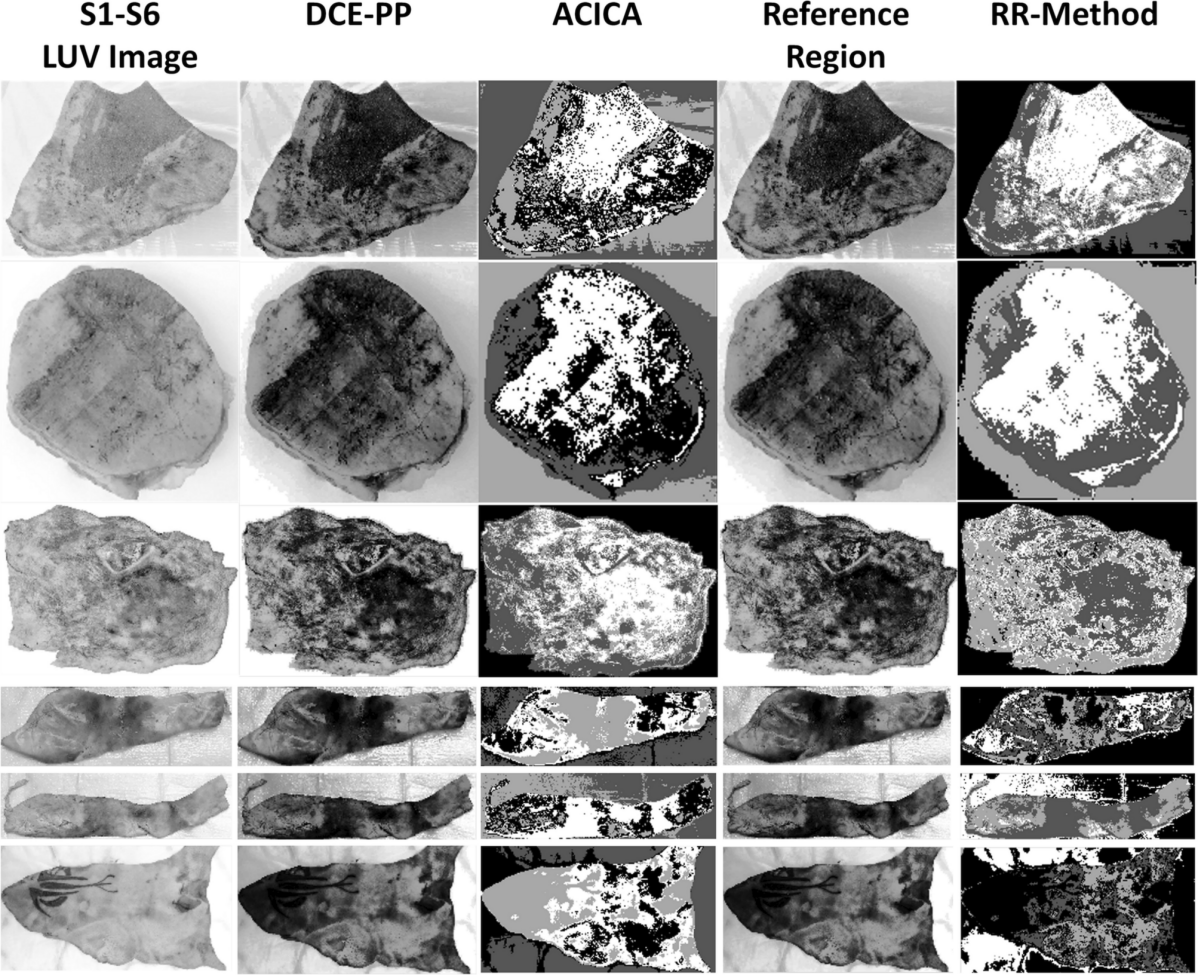
Comprehensive Analysis of Burn Samples (S1-S6) Using Various Imaging Techniques.

In this comprehensive analysis, each sample (S1 through S6) undergoes detailed examination using various imaging and processing techniques, presented across multiple columns in Figure 7. Each column represents a specific method used to enhance^65^ and analyze the burn images, providing a multifaceted view of the burn areas and facilitating a thorough understanding of burn severity and distribution.

Column 1 (LUV Images): The first column showcases the LUV images of each sample, representing the luminance (L) and chromaticity (UV) components. The LUV color space is chosen for its perceptual uniformity, which ensures consistent color perception critical for accurate burn analysis. These images serve as the foundational visual data, from which further processing techniques are applied to enhance and analyze the burn areas.

Column 2 (DCE-PP (Dynamic Contrast Enhancement with Perceptual Processing)): The second column presents images processed using the DCE-PP technique. This method enhances the contrast of the images, making it easier to distinguish between different burn depths. By highlighting subtle variations in tissue properties, the enhanced contrast allows for a more detailed visual inspection of the burn areas, aiding in the identification of minor but clinically significant differences.

Column 3 (ACICA): The third column features images processed with ACICA. This advanced technique is known for its ability to decipher complex data relationships and extract relevant features from burn images. ACICA significantly enhances the accuracy of burn depth analysis, providing a detailed understanding of the burn severity and the structural changes within the tissue. The method is particularly effective in separating overlapping features, which is crucial for accurate diagnosis.

Column 4 (Reference Region): The fourth column displays the Reference Region images. Here, areas with different burn depths are outlined by software using algorithms to detect color differentiations. This segmentation is critical for dividing the burn areas into distinct regions based on the severity of the injury. The outlined regions serve as a guide for further analysis and comparison with other processing methods, ensuring a standardized approach to evaluating the burn severity.

Column 5 (RR-Method (Region Recognition Method)): The fifth column illustrates the results obtained using the RR-Method. This technique focuses on accurately identifying and isolating specific areas within the burn images. By concentrating on distinct regions, the RR-Method allows for a more detailed and precise analysis of the extent and nature of the burns. The method’s focus on region-specific characteristics enhances the granularity of the assessment, making it possible to identify variations within the same burn area.

Each sample’s figure 7 set, encompassing these five columns, delivers a multi-dimensional analysis of burn injuries. This approach integrates various advanced image processing techniques, enhancing diagnostic accuracy and providing a comprehensive view of the burn severity and distribution across different regions. The combination of these methods offers valuable insights into the nature of the burns, facilitating better-informed decisions in clinical settings and advancing the understanding of burn injury assessment.

### Statistical analysis and visualization

**Fig. 8:**
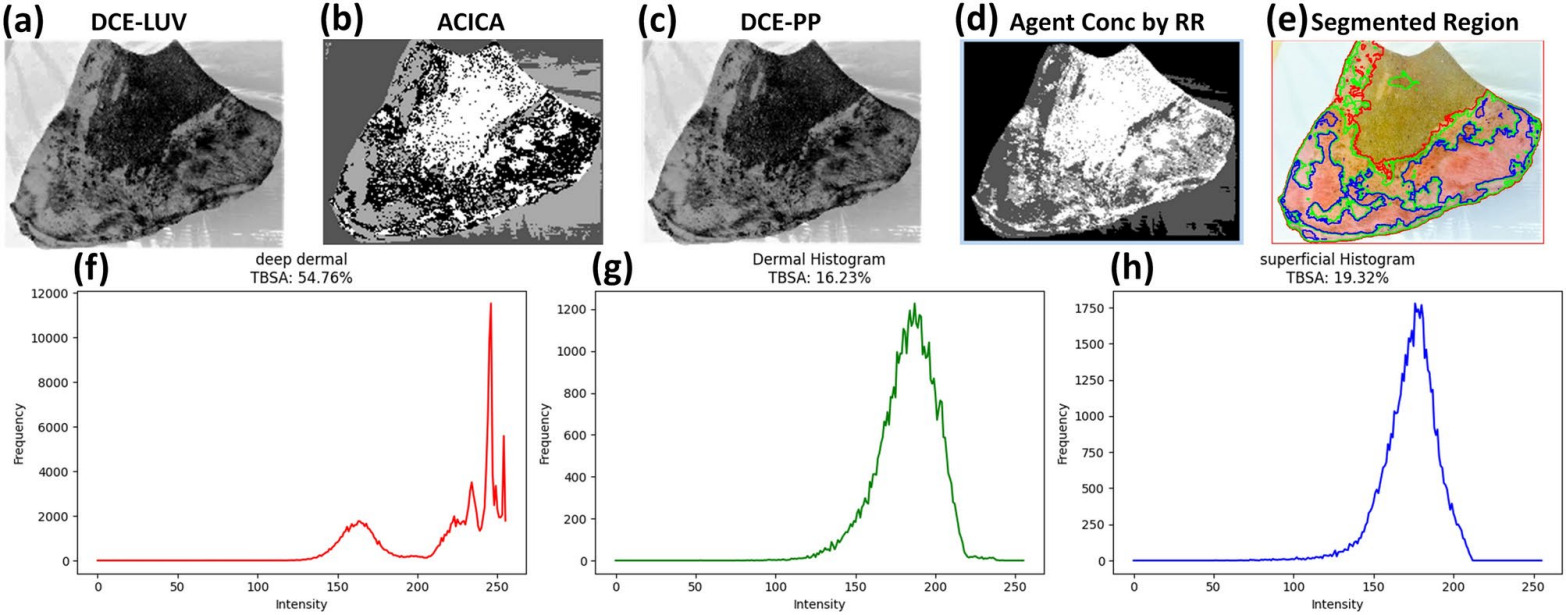
Comprehensive Analysis and Segmentation of Burn Samples: (**a**) DCE sample demonstrating enhanced statistical parameters. (**b**) ACICA method evaluating the concentration of contrast agents in burn tissue. (**c**) Application of the Reference Region (RR) method. (**d**) RR threshold facilitates peak clustering in close proximity for precise identification. (**e**) Burn sample segmentation using a Deep Neural Network (DNN). (**f**) Histogram analysis of the deep dermal burn image. (**g**) Histogram examination of the dermal region. (**h**) Histogram of the superficial burn image.

In Figure 8(a), we present a DCE sample that showcases enhanced statistical parameters. This sample illustrates the advancements achieved in analyzing and representing statistical data within medical images. The term “Statistical Parameters” refers to various metrics such as mean, standard deviation, entropy, skewness, and kurtosis, which characterize the distribution of contrast agents within the image. Enhancements in these parameters provide more accurate and informative insights, contributing to the refinement of medical image analysis and diagnosis. Figure 8(b) presents an example of the output generated by the Adaptive Complex Independent Components Analysis (ACICA) method, used to evaluate the concentration of contrast agents in burn tissue. The ACICA algorithm calculates the intra-components and converts the signal from the patterns into a curve representing the concentration of the contrast agent. This algorithm effectively addresses the issue of underestimating the pattern during the early stages of contrast agent uptake, as it does not rely on prior knowledge of contrast distribution in burns outside the regular region.

Figure 8(c) illustrates the application of an RR-based technique for correcting the tissue concentration curve obtained through the ACICA method. This correction is achieved through clustering and thresholding mechanisms. Values falling beneath a specific threshold are identified and removed from the cluster centers, enhancing precision. To provide comprehensive visualization, the histogram and refined cluster centers are presented together, contributing to a more insightful understanding of their interrelationships.

Figure 8(d) examines the pixel distribution of the Reference Region (RR). This step involves creating a quantized version of the image to understand the spread of pixel intensities better. The threshold “Dth” is used to facilitate the clustering of closely situated peaks, ensuring precise identification. Figure 8(e) demonstrates burn sample segmentation using an RNN network, which outperforms FNN and CNN. The examination of ACICA and RR approaches reveals that using these techniques enhances the features of the burn sample, resulting in improved burn profile segmentation. GLCM characteristics such as contrast and correlation further improve segmentation accuracy. The visual output includes the original image, an image with segmented parts, and histograms. The segmented image shows the burn locations marked in various colors.

Figures 8(f), 8(g), and 8(h) represent the steps of our histogram analysis and peak identification procedure. First, the “imhist” function creates a histogram of the image’s pixel intensity distribution. The “zth” and “Dth” thresholds are then applied to reduce dark pixels and improve peak detection accuracy. These processes ensure noise-free analysis and reliable detection of crucial intensity levels, indicating distinct tissue properties. The histograms depict the intensity distribution for superficial, superficial dermal, and deep dermal burns, aiding in the examination of burn features. Comprehensive visualization and quantification are critical for the medical assessment and treatment planning of burn wounds.

The technique estimates the regions covered by each type of burn and their percentages relative to the overall image area, also known as total body surface area (TBSA) percentages. These TBSA percentages provide a quantitative assessment of the severity of each burn type. Additionally, histograms for each burn type are examined, displaying the distribution of L values within the LUV color space. These histograms graphically represent the frequency of pixel intensities, helping differentiate burn types based on their intensity patterns.

#### Performance comparison of ACICA and RR methods

The ACICA and RR methods demonstrate superior performance across various metrics, as summarized in Table 3 and illustrated in Figure 8. These methods consistently outperform traditional approaches in segmentation accuracy, burn depth classification, and TBSA estimation.

**Table 3:** Performance Metrics for Segmentation, Classification, and TBSA Estimation.

**Method**	**Segmentation accuracy (%)**	**Classification F1-Score**	**TBSA estimation error (%)**
ACICA^26^	92.5	0.91	4.3
RR Method^66^	90.7	0.89	5.1
Traditional Methods	82.3	0.78	12.8

The quantitative results in Table 3demonstrate that ACICA achieves the highest segmentation accuracy (92.5%) and classification F1-score (0.91)^67^, with minimal TBSA estimation error (4.3%)^68^. The RR method also performs well, achieving a segmentation accuracy of 90.7% and a classification F1-score of 0.89^69^. In contrast, traditional methods exhibit significantly lower performance across all metrics^70^, with a segmentation accuracy of 82.3%, classification F1-score of 0.78, and TBSA estimation error of 12.8%.

Figure 8 provides a visual comparison of the segmentation outcomes. The delineation of burn regions using ACICA and RR methods is visibly superior to that achieved by traditional techniques. This improvement highlights the ability of the proposed methods to capture fine details and complex tissue structures, which are critical for accurate burn assessment.

### Performance of deep learning models

**Fig. 9:**
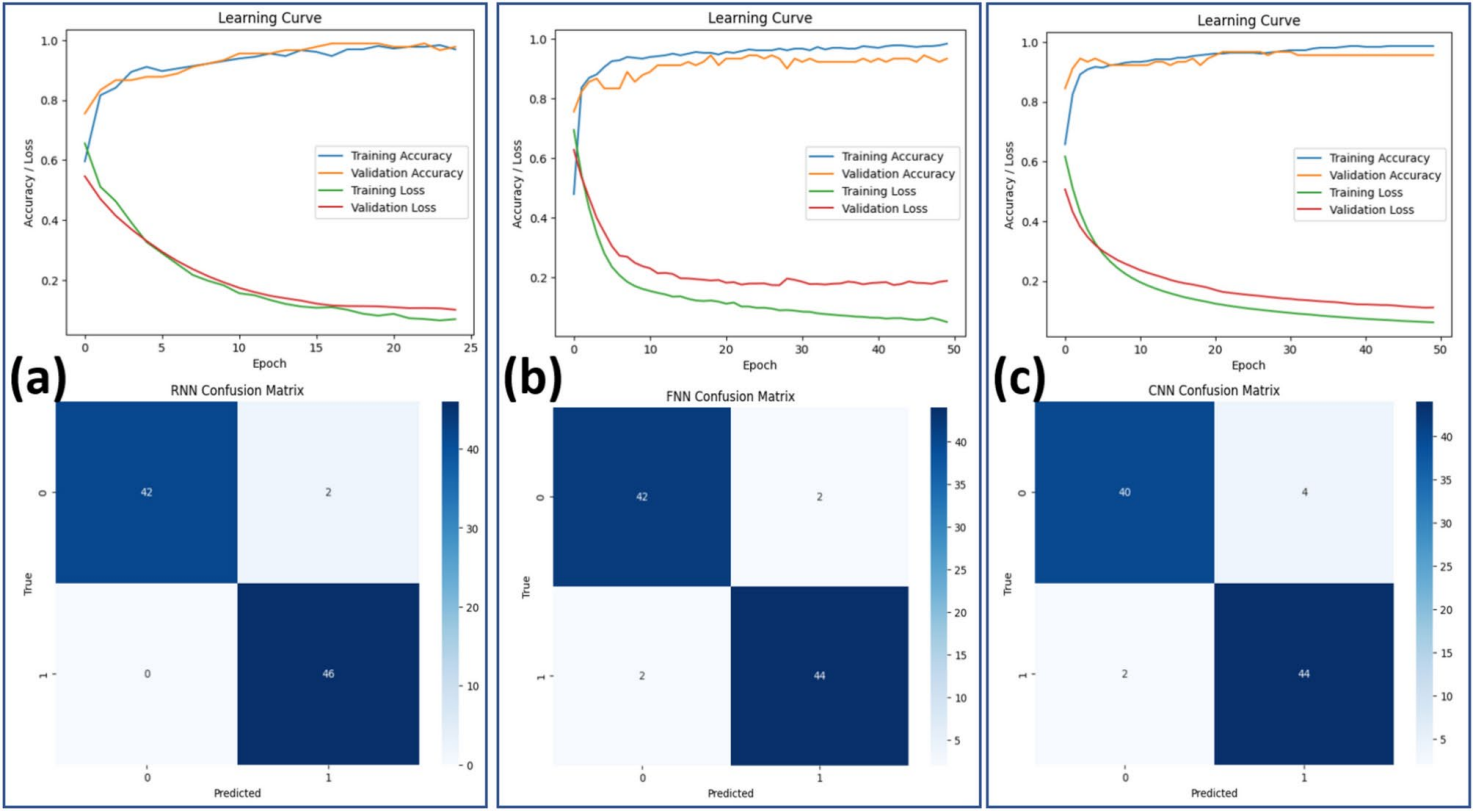
Performance Analysis of Deep Learning Models for Burn Injury Prediction (**a**) Performance analysis of the Recurrent Neural Network (RNN) model for burn injury prediction, showcasing accuracy and loss curves, and the confusion matrix. (**b**) Evaluation of the Feedforward Neural Network (FNN) model for burn injury prediction, presenting accuracy and loss curves, and the confusion matrix. (**c**) Assessment of the Convolutional Neural Network (CNN) model for burn injury prediction, including accuracy and loss curves, and the confusion matrix.

These analyses utilize a dataset of 90 images to evaluate and compare the effectiveness of the three neural network architectures-RNN, FNN, and CNN-in predicting burn injuries. Figure 9 each subplot illustrates the training and validation accuracy and loss over epochs, alongside the confusion matrix, which visually summarizes the model’s performance by depicting the true versus predicted classifications. This comprehensive evaluation helps in understanding the strengths and limitations of each model in the context of burn injury diagnosis.

Figure 9(a) illustrates The RNN model performance with an astounding accuracy of 96.67%. This accuracy measure shows the percentage of correctly identified cases among all the analyzed cases. In addition, the model shows strong recall, precision, and F1-score values for class 0 and class 1, respectively, of 0.96, 0.98, and 0.97 and 0.95, 0.97, and 0.97, respectively. Recall assesses how well the model can identify positive occurrences among all real positive instances, whereas precision measures how well the model can identify positive events among all anticipated positive instances. The F1 score is a balanced statistic that takes into account both false positives and false negatives. It is calculated as the harmonic mean of precision and recall. These excellent recall, precision, and F1-score results imply that the model successfully reduces both erroneous negative and positive forecasts. Furthermore, the model’s general efficacy across classes is further validated by the macro average and average weighted metrics, which are both computed as 0.97 for accuracy, recollection, and F1-score. This highlights the model’s reliability and consistency in diabetes prediction.

Figure 9(b) illustrates The FNN model performance achieving an impressive accuracy of 95.56%. This accuracy metric indicates the percentage of correctly predicted cases among all instances analyzed. Additionally, the model displays commendable recall, precision, and F1-score values for both class 0 and class 1, with values of 0.95, 0.95, and 0.95 for class 0, and 0.96, 0.96, and 0.96 for class 1, respectively. Recall evaluates the model’s ability to identify positive occurrences among all actual positive instances, while precision assesses its accuracy in identifying positive events among all predicted positive instances. The F1-score, being a balanced statistic, considers both false positives and false negatives. These strong recall, precision, and F1-score outcomes suggest that the model effectively minimizes both false negative and false positive predictions. Furthermore, the model’s overall effectiveness across classes is validated by the macro average and weighted average metrics, both of which calculate to 0.96 for accuracy, recall, and F1-score. This underscores the model’s reliability and consistency in predicting burn injuries. Overall, these performance metrics underscore the FNN model’s potential utility in early detection and management strategies within clinical and healthcare settings, validating its efficacy in accurately forecasting burn patients.

In the given binary classification problem, the Convolutional Neural Network (CNN) model demonstrated in Figure 9(c) shows the ability to accurately categorize instances from the dataset with an excellent accuracy of 93.33%. The model’s performance for each class is further clarified by looking at the accuracy, recall, and F1-score metrics. In class 0, the recall of 0.91 suggests that the model accurately recognized 91% of the real class 0 cases, while its precision of 0.95 shows that 95% of examples categorized as class 0 were right. For class 0, the F1-score of 0.93 indicates a harmonic mean of accuracy and recall that is balanced. Comparably, for class 1, the recall of 0.96 shows that the framework correctly recognized 96% of the examples, while the precision of 0.92 implies 92% accuracy in categorizing instances as class 1. Class 1’s F1-score of 0.94 indicates that recall and accuracy are harmoniously balanced. The model’s resilience in classification tasks is confirmed by the macro average and average weighted metrics, which both consistently show a balanced score across all classes at 0.93. All things considered, these findings show that the CNN model performs well, reliably and accurately differentiating between examples of class 0 and class 1.

#### RNN model

The RNN model performs well in burn profile prediction, with an accuracy of 96.67%. The model’s recall, precision, and F1-score for class 0 and class 1 are 0.96, 0.98, 0.97, 0.95, 0.97, and 0.97, respectively. These metrics indicate that the model effectively minimizes both false negative and false positive predictions. The macro average and weighted average metrics, both 0.97, further validate the model’s reliability and consistency.

#### FNN model

The FNN model achieves an accuracy of 95.56%, with recall, precision, and F1-score values for class 0 and class 1 being 0.95, 0.95, 0.95, 0.96, 0.96, and 0.96, respectively. The model’s macro average and weighted average metrics are 0.96, highlighting its reliability and consistency.

#### CNN model

The CNN model demonstrates an accuracy of 93.33%. For class 0, the recall is 0.91, the precision is 0.95, and F1 score is 0.93. For class 1, the recall is 0.96, the precision is 0.92, and F1 score is 0.94. The model’s macro average and weighted average metrics are both 0.93, confirming its robustness in classification tasks.

**Table 4:** Model Performance Comparison.

**Model**	**Acc**	**Preci**	**Recall**	**F1-S**	**PT(s)**
RNN	0.967	0.97	0.97	0.97	0.240
FNN	0.956	0.96	0.96	0.96	0.087
CNN	0.933	0.93	0.93	0.93	0.107

Table 4 summarizes the performance and prediction efficiency of the RNN, FNN, and CNN models. The RNN model shows the highest accuracy (96.7%) but with a longer prediction time (0.240 seconds). The FNN model follows closely with an accuracy of 95.6% and the fastest prediction time (0.087 seconds). The CNN model, while slightly behind in accuracy (93.3%), still performs well with a prediction time of 0.107 seconds.

**Fig. 10:**
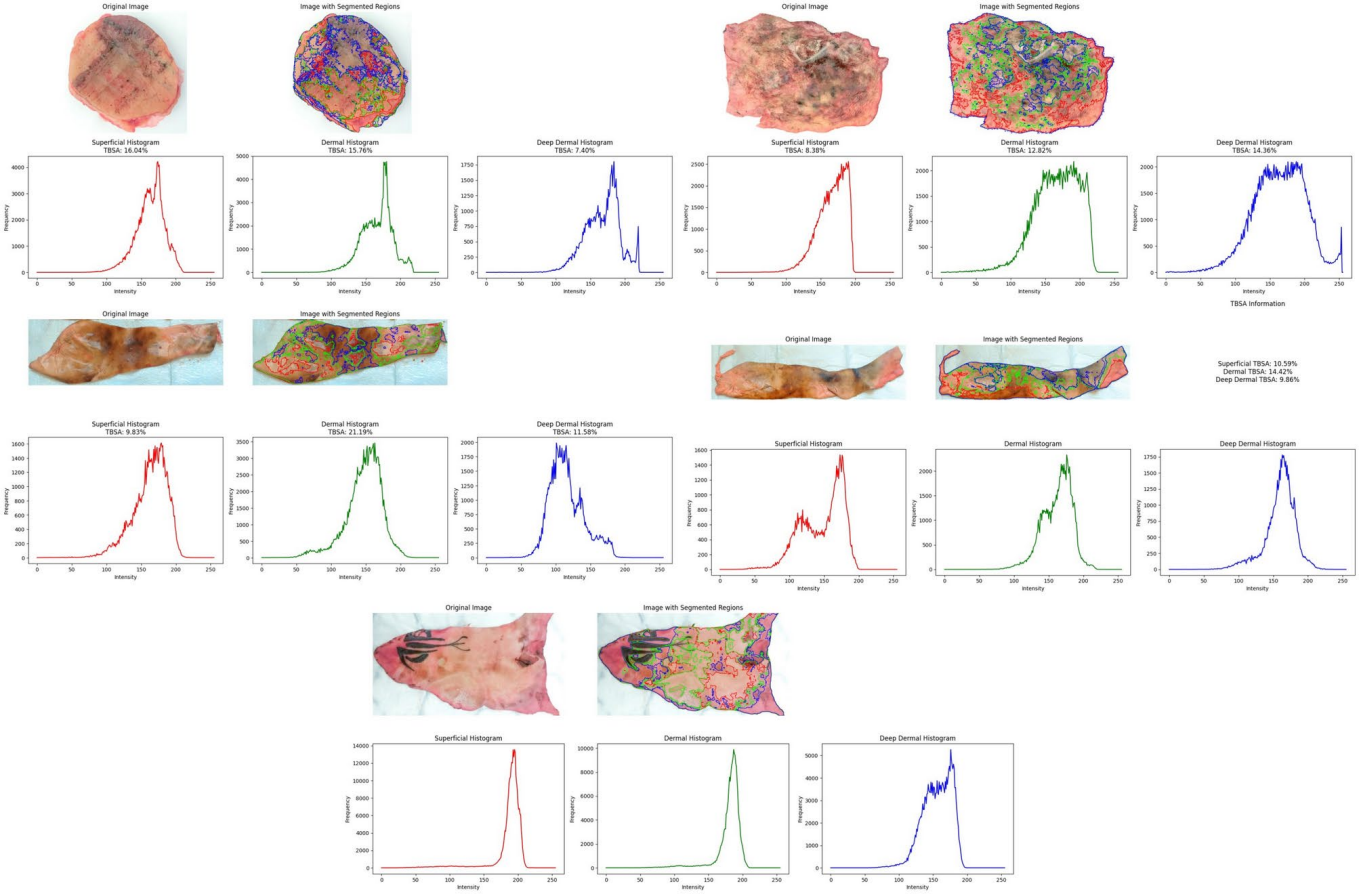
Comprehensive Analysis of Burn Injuries.

In this detailed exploration of burn injuries, each sample receives thorough analysis through a series of figures (Figure 10), offering nuanced insights into the nature and severity of the burns. Each sample’s figure set comprises distinct components: the Original Image, Image with Segmented Regions, Total Body Surface Area (TBSA) percentages for specific burn depths, and Histograms illustrating burn distribution. The Original Image serves as the unaltered visual depiction of the burn area, capturing the initial condition of the tissue samples. This unprocessed image provides a baseline for subsequent analyses, offering an unadulterated view of the visible injuries present in each sample. Utilizing advanced processing techniques like the Grey-Level Co-occurrence Matrix (GLCM), the Image with Segmented Regions is meticulously generated. This segmented image utilizes sophisticated algorithms to color-code and delineate various burn depths, enabling precise visualization of affected regions specific to each sample. TBSA percentages are visually represented through pie charts, categorizing burns into superficial, superficial dermal, and deep dermal layers. The distinct color scheme employed facilitates immediate comprehension of burn distribution and severity within each sample, enabling a comprehensive assessment tailored to individual characteristics. The Histogram offers a statistical overview of burn distributions by depicting the frequency of pixel intensities across different burn depths. This quantitative analysis provides nuanced insights into texture variations within each burn depth category, complementing the visual segmentation provided by the Image with Segmented Regions. Each sample’s figure set is accompanied by a detailed analysis, referencing specific features observed within the tissue samples. For instance, in Sample S1, severe burn injuries are predominantly categorized as Deep burns, supported by both the Image with Segmented Regions and the TBSA percentages. Sample S2 exhibits moderate burn injuries with a balanced distribution of Superficial and Dermal burns. Sample S3 displays mild to moderate burn injuries with an even distribution among superficial, superficial dermal, and deep dermal burns. Sample S4 showcases distinct burn injuries with a higher prevalence of Dermal burns. Sample S5 demonstrates moderate to severe burn injuries with a balanced distribution of burn depths. Comprehensive instructions accompany the figures (Figure 10), guiding users in interpreting the images and selecting the corresponding skin condition or degree of burn injury. Additionally, a disclaimer emphasizes compliance with data protection regulations, ensuring anonymity and confidentiality in data collection and usage. Overall, this approach delivers a scientifically rigorous and comprehensive analysis of burn injuries, facilitating informed decision-making in clinical settings and advancing understanding in the field of burn injury assessment.

### Clarification on the use of TBSA in burn sample analysis

In clinical burn care, TBSA is a critical metric used to estimate the extent of burn injuries relative to the patient’s entire skin surface. For instance, if an entire arm is burned, it is generally considered to represent approximately 9% of the patient’s TBSA. Thus, a patient with a fully burned arm would be reported as having 9% TBSA affected.

In this study, however, the term TBSA is employed in a specific context distinct from its conventional clinical application. Here, TBSA refers to the percentage of the burned area within an individual sample, with the entire sample being considered 100% of the burned area for analytical purposes. For example, if a burn sample is analyzed and found to have 90% of its area affected by deep burns and 10% by superficial burns, we denote this as 90% deep TBSA and 10% superficial TBSA within that sample. It is important to note that this application of TBSA pertains solely to the individual burn samples examined and does not reflect the patient’s overall TBSA. In this context, the TBSA percentages reported are relative to the specific sample rather than the patient’s total body surface.

To avoid confusion and ensure clarity, we will clearly state this methodological distinction in our manuscript. This approach allows for a detailed analysis of burn depth within each sample, although it deviates from traditional clinical usage of TBSA. We acknowledge that this non-standard application of TBSA might be unconventional, and we aim to provide this clarification to prevent any potential misinterpretation by clinicians and other professionals in the field.

### TBSA estimation

Each sample’s Figure 7 set, encompassing these five columns delivers a multi-dimensional analysis of burn injuries. This approach integrates various advanced image processing techniques, enhancing diagnostic accuracy and providing a comprehensive view of the burn severity and distribution across different regions. The combination of these methods offers valuable insights into the nature of the burns, facilitating better-informed decisions in clinical settings and advancing the understanding of burn injury assessment.

**Fig. 11:**
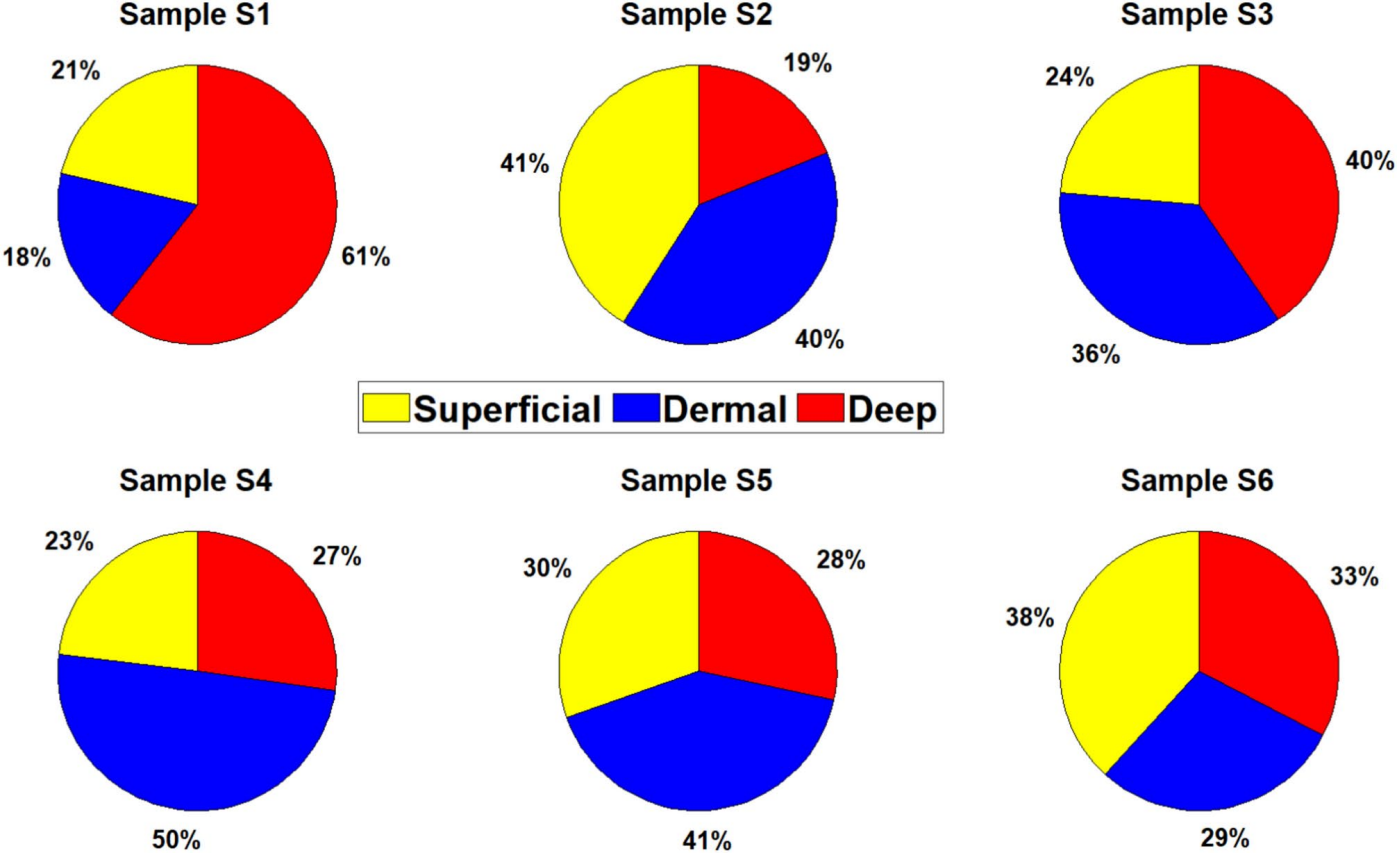
Pie charts illustrating the Total Body Surface Area (TBSA) percentages for six samples (S1 to S6), categorized into Superficial (yellow), Dermal (blue), and Deep (red) burns. Each pie chart represents the distribution of burn depths within a specific sample, providing a visual understanding of the severity and extent of burns.

Figure 11 showcases the Total Body Surface Area (TBSA) percentages for six different samples (S1 to S6), with each sample divided into three categories of burns: superficial, superficial dermal, and deep dermal. The colors yellow, blue, and red correspond to superficial, superficial dermal, and deep dermal burns, respectively. This visual representation is crucial for understanding the distribution and severity of burns across different samples, thereby aiding in the assessment and treatment planning for burn injuries.

The data for each sample was obtained through a detailed analysis of burn images using the Grey-Level Co-occurrence Matrix (GLCM) technique. This technique was applied to decomposed luminance and color components to extract essential texture features, namely contrast and correlation. These features were computed using an 8$\times$8 mask with offsets at 0, 45, 90, and 135 degrees to ensure comprehensive texture analysis across various orientations. This approach ensures that the texture features are captured accurately from different angles, enhancing the reliability of the extracted data.

Each sample’s TBSA percentages are visualized using pie charts, providing a clear and immediate understanding of the distribution of different burn depths within each sample. The chosen color scheme (yellow for Superficial, blue for Dermal, red for Deep) allows for distinct and easy-to-interpret visualization, with each segment representing the proportion of a specific burn type within the total body surface area. The visual representation highlights the severity and extent of burns, enabling effective identification and delineation of burn regions.

Sample S1 represents a moderate burn injury with a predominant distribution of deep dermal burns, accounting for 54.76% of the total body surface area (TBSA). This sample’s analysis reveals significant structural changes in the deeper layers of the tissue, as highlighted by the ACICA. Sample S2 showcases a balanced distribution of burn depths, with 16.04% superficial, 15.76% dermal, and 7.40% deep burns. The DCE-PP and RR-Method images particularly highlight the subtle differences in tissue properties, facilitating a comprehensive understanding of the burn severity.

Sample S3 displays mild to moderate burn injuries with a relatively even distribution among the burn depths: 8.38% superficial, 12.8% dermal, and 14.36% deep burns. The segmented regions and histograms indicate a mixture of tissue damage levels, emphasizing the importance of using multiple techniques for accurate analysis. Sample S4 reveals distinct burn injuries with a higher prevalence of dermal burns (21.19%). The ACICA images provide a detailed view of the burn severity, while the histograms illustrate the distribution of pixel intensities across different burn depths.

Sample S5 demonstrates moderate to severe burn injuries with a balanced distribution of burn depths: 10.59% superficial, 14.42% dermal, and 9.86% deep burns. The enhanced contrast and segmented regions highlight the variations within the burn areas, offering valuable insights into the tissue damage. Sample S6 shows significant burn injuries with 20.46% superficial, 15.54% dermal, and 17.43% deep burns. The combination of ACICA and RR-Method techniques provides a comprehensive analysis of the burn severity, while the histograms further quantify the texture variations within the burn areas.

This Figure 11 effectively encapsulates the burn severity and distribution across different samples, offering critical insights for clinical assessment and intervention planning. The use of advanced image processing techniques, such as GLCM, ensures the accuracy and reliability of the extracted features, thereby enhancing the overall diagnostic process.

### Comparison with recent methods

To evaluate the effectiveness of the proposed framework, its performance was compared with several state-of-the-art methods for burn injury classification. Table 5 summarizes the accuracy achieved by various approaches reported in recent literature, alongside the accuracy of our proposed system.

**Table 5:** Comparison of Classification Accuracy with Recent Methods.

**Study**	**Accuracy (%)**
Chauhan et al.^71^ (2020)	92.5
Rostami et al.^72^ (2021)	88.2
Abubakar et al.^73^ (2020)	95.43
Rahman et al.^74^ (2022)	87.50
**Proposed System**	**96**.**70**

The study by Chauhan and Goyal (2020) proposed the Body Part-Specific Burn Severity Assessment Model (BPBSAM), achieving an accuracy of 92.5% using a hybrid approach combining deep learning^75^and region-specific analysis for burn images^71^. While effective, the method focuses on specific body parts, potentially limiting its generalizability across varied datasets. Rostami et al. (2021) employed deep convolutional neural networks for multiclass burn wound image classification, reporting an accuracy of 88.2%. Despite its success, the approach demonstrated challenges in handling highly heterogeneous datasets, leading to moderate classification performance^72^.

Abubakar et al. (2020) utilized deep transfer learning with ResNet50 and VGG16 models, achieving an accuracy of 95.43% on a dataset of 2080 RGB images. Although this method achieved competitive results, its reliance on pre-trained models and large datasets underscores the need for more compact and scalable frameworks^73^. Rahman et al. (2022) developed a CNN-based model focusing on burn inflammation assessment, achieving an accuracy of 87.50%. This approach, while relevant to inflammation-specific studies, exhibits limitations in general burn classification due to its narrow focus on inflammatory markers^74^.

The proposed system achieved an accuracy of 96.70%, outperforming all other methods in the comparison. By integrating advanced techniques such as ACICA, RR segmentation, and texture-based GLCM analysis, the framework demonstrated superior performance in burn depth classification and TBSA estimation. The combination of these robust methods with a well-optimized CNN model contributed to its high classification accuracy, even with a relatively smaller dataset of 90 images. This comparative analysis highlights the efficacy of the proposed system in achieving state-of-the-art performance for burn injury assessment. Its superior accuracy, combined with computational efficiency, underscores its potential for integration into clinical workflows.

## Conclusion

The proposed methodology demonstrates high effectiveness in analyzing burn wounds against complex backgrounds with notable accuracy. The ACICA and RR methods exhibit robustness, independent of camera resolution, by operating within the LUV color model instead of relying on RGB images. This characteristic ensures consistent performance across various camera resolutions. The proposed method achieves significant data reduction without compromising information integrity, particularly in the estimation of image sources, making it highly suitable for real-time processing.

In contrast to PCA and ICA, which often show inconsistent performance in feature analysis across different burn images and can lead to over/under-segmentation errors, the ACICA and RR methods offer distinct advantages. ACICA adapts dynamically to contrast variations, enhancing its adaptability to diverse image conditions. Additionally, RR provides robust regression capabilities, contributing to more stable and reliable results compared to PCA and ICA.

We conducted a robust quantitative assessment using various statistical parameters, ensuring an unbiased evaluation of improved image contrast. Our approach efficiently retrieves relevant medical images through innovative techniques like Adaptive Complex Independent Components Analysis, Curve Evaluation and Correction, and the incorporation of GLCM. Extensive experimentation on a dataset comprising DCE-LUV samples validates the effectiveness of our approach. The DCE-LUV images exhibit a substantially higher mean (76.875) compared to the query image (56.65), indicating enhanced contrast enhancement. Furthermore, differences in standard deviation (query image: 18.24, DCE-LUV image: 15.35), higher entropy (query image: 5.68, DCE-LUV image: 6.25), skewness (query image: 2.05, DCE-LUV image: 2.56), and kurtosis (query image: 125.69, DCE-LUV image: 169.35) values highlight variations in agent distribution complexity and asymmetry, enhancing diagnostic precision.

In terms of accuracy, precision, recall, and F1-Score, the RNN model outperforms the FNN and CNN models, achieving 96.7% accuracy and F1-Score, respectively, with a precision and recall rate of 97.0%. Although it has a slightly higher prediction time (0.240 seconds) than FNN and CNN, its superior performance makes it an appealing choice for tasks requiring high accuracy. The FNN model follows closely with an accuracy of 95.6% and an F1-Score of 96.0%, and it has the shortest prediction time of the three models (0.087 seconds), making it ideal for real-time applications. While the CNN model trails slightly with an accuracy of 93.3% and F1-Score, it still performs well and has a forecast time of 0.107 seconds.

Our approach significantly improves scores for top-matching image features from the database, emphasizing its practical utility in medical image analysis and diagnosis. The integration of a deep neural framework utilizing visual descriptors further augments diagnostic precision, empowering clinicians with invaluable insights for informed decision-making in patient care.

Our research not only advances burn profile diagnosis and treatment but also holds the potential to revolutionize medical image analysis, ultimately leading to improved patient outcomes and more efficient healthcare practices. This work paves the way for future innovations in the field, promising significant enhancements in clinical diagnostics and patient care.

## Data Availability

The datasets generated and analyzed during the current study are not publicly available due to ethical and privacy considerations. These datasets include sensitive patient information and were subject to strict data protection protocols as part of the ethical approval process. However, data may be made available from the corresponding author, Robin Augustine, upon reasonable request, provided that the request complies with ethical guidelines and data protection regulations.
